# Beyond Molecular Classification: The Incremental Value of Prognostic Biomarkers in Endometrial Cancer

**DOI:** 10.3390/jcm15208154

**Published:** 2026-10-20

**Authors:** Vlad Gabriel Tirnovanu, Iustina Petra Solomon Condriuc, Raluca Anamaria Mogos, Daniela Roxana Matasariu, Alexandru Filipescu, Mihaela Burac, Mihaela Camelia Tirnovanu, Demetra Socolov, Alexandru Carauleanu

**Affiliations:** 1 Grigore T. Popa University of Medicine and Pharmacy, 700115 Iasi, Romaniademetra.socolov@umfiasi.ro (D.S.); ale.carauleanu@umfiasi.ro (A.C.); 2 Department of Obstetrics and Gynecology, Cuza Voda Hospital, 700038 Iasi, Romania; 3 Department of Obstetrics and Gynecology, “Carol Davila” University of Medicine and Pharmacy, 8 Eroii Sanitari Blvd., 050474 Bucharest, Romania; 4 Department of Obstetrics and Gynecology, Elias University Emergency Hospital, 17 Mărăști Blvd., 011461 Bucharest, Romania; 5 Department of Obstetrics, Gynecology and Human Reproduction, “Nicolae Testemițanu” State University of Medicine and Pharmacy, MD-2004 Chișinău, Moldova

**Keywords:** endometrial cancer, molecular classification, tumor biomarkers, prognosis, estrogen receptor, L1 cell adhesion molecule, risk stratification, prediction models

## Abstract

Molecular classification underpins prognostic assessment in endometrial carcinoma, but heterogeneity persists within molecular classes. This narrative review examines whether tissue-based, circulating and emerging biomarkers add prognostic information beyond molecular class and clinicopathological factors. We distinguish four evidentiary levels: prognostic association, independence after adjustment for molecular class, within-class stratification and incremental predictive performance. Clinical utility is considered separately. Estrogen receptor (ER) status now contributes to risk stratification of no specific molecular profile (NSMP) tumors in the 2025 European guidelines, primarily on within-class prognostic evidence. Its incremental predictive performance has been demonstrated only in high-risk disease, with internal validation. Clinical utility remains unproven, and findings within mismatch repair-deficient disease are discordant. *CTNNB1* mutation has shown inconsistent associations with recurrence, mainly in early-stage disease. Chromosome 1q32.1 amplification was associated with recurrence within NSMP in two cohorts. Neither marker has been formally assessed for incremental predictive performance. Where model comparisons were performed, gains in discrimination were generally modest. Larger gains arose from small samples or models without external validation, and external validation remained uncommon. No individual biomarker examined has demonstrated prospective clinical utility on its own.

## 1. Introduction

Endometrial carcinoma is the most frequently diagnosed gynecological malignancy in high-income countries [1], and its incidence is rising [2]. Prognostic assessment governs the central decision in early-stage disease: whether to give adjuvant treatment after surgery. For most of the twentieth century, it rested on histological type, grade, myometrial invasion, lymphovascular space invasion (LVSI) and stage, a framework that is only partly reproducible [3].

The Cancer Genome Atlas (TCGA) reorganized the disease into four groups defined by *POLE* exonuclease domain mutation, microsatellite instability and somatic copy-number burden, with frequent *TP53* mutation in the copy-number-high group [4]. Immunohistochemical surrogates combined with targeted *POLE* sequencing reproduced the survival separation at a cost compatible with routine pathology [5,6]. The resulting classes are *POLE*-ultramutated (POLEmut), mismatch repair-deficient (MMRd), no specific molecular profile (NSMP) and p53-abnormal (p53abn). They entered the 2020 World Health Organization classification and the 2021 European guidelines [7,8], then the 2023 International Federation of Gynecology and Obstetrics (FIGO) staging system and the 2025 European update [9,10]. Molecular class is now the reference against which any new prognostic marker must be judged.

Classification did not eliminate variation in outcome within each class. NSMP is the clearest illustration. It is defined by exclusion rather than by a shared driver, and it is the largest of the four classes. It accounted for 50.4% of 452 tumors in a population-based validation cohort [11]. In high-grade endometrioid disease, pooled overall survival for NSMP tumors falls between that of POLEmut and p53abn disease, and neither comparison reaches significance [12]. Heterogeneity is not confined to NSMP, and its determinants differ by class. Even LVSI, a long-established variable, predicts progression at different thresholds in MMRd and NSMP tumors, whereas neither threshold showed a statistically significant association in the smaller p53abn subgroup [13].

Residual risk of this kind is what a second-tier biomarker might explain. Most biomarker studies asked whether a marker is associated with outcome, or whether it retains significance alongside stage and grade. Neither is the question that now matters, which is whether the marker adds information to a model already containing molecular class. Statistical independence and incremental value are distinct properties, formalized for two decades in the literature on tumor-marker reporting and prediction-model evaluation [14,15].

Recent systematic reviews have assessed candidate prognostic modifiers specifically within NSMP disease, with searches closing before estrogen receptor-based refinement entered the 2025 European framework [16,17]. The present review asks a different question. It examines whether additional biomarkers provide prognostic information beyond contemporary molecular classification itself, across molecular subgroups and across tissue-based, circulating and emerging biomarker domains. Throughout, we separate four evidentiary levels: prognostic association, independence after adjustment for molecular class, within-class prognostic stratification, and incremental predictive performance. Clinical utility is treated as a further translational requirement rather than as an automatic consequence of improved prediction.

### Approach to the Literature

This is a narrative review. We identified literature through PubMed/MEDLINE, Scopus and Web of Science. Terms for endometrial carcinoma and molecular classification were combined with terms for prognostic biomarkers and risk stratification, and with individual marker names. Literature identification was extended by tracking citations from key papers. The narrative synthesis focused on studies directly relevant to the review question. The literature considered covered tissue-based, circulating and emerging biomarkers (Section 4, Section 5 and Section 6), together with methodological work on tumor-marker reporting and prediction-model evaluation (Section 3). Because the question concerns what a marker adds once molecular class is known, preference was given to molecularly annotated cohorts, translational analyses of randomized trials, recent meta-analyses and current guidelines. These sources can show whether an association persists after adjustment for molecular class or holds within a single class, and whether a marker improves a model that already contains molecular class, which are the distinctions drawn in Section 3. Earlier studies that predate molecular classification were considered where they established the original prognostic association against which later, molecularly adjusted findings are read.

A candidate biomarker was prioritized for discussion if molecularly annotated outcome data existed for it. It was also considered if it had been evaluated within molecular subgroups, in a contemporary validation cohort, in a recent meta-analysis, or in a current risk-stratification framework. The evidence for each biomarker discussed was then placed on the four levels defined in Section 3, using the assignment rules given there. Where findings conflicted, discordant studies were considered and are presented alongside supportive ones, particularly for L1 cell adhesion molecule (L1CAM), *CTNNB1* mutation and estrogen receptor status outside NSMP, rather than the conflict being resolved by selective citation. Coverage is purposeful rather than exhaustive. Candidates that met none of the conditions above were not discussed individually, so the review does not cover every marker reported as prognostic in endometrial cancer. Given the narrative design, no formal risk-of-bias assessment or quantitative synthesis was performed. Search strings, numbers of records screened and formal eligibility criteria are not reported: the account given here is meant to make the approach transparent, not to allow the identification of studies to be reproduced as in a systematic review. Reporting was informed by the criteria of the Scale for the Assessment of Narrative Review Articles (SANRA) [18], and an item-by-item assessment of this review against them is provided in Supplementary Table S1.

## 2. Molecular Classification as the Prognostic Baseline

### 2.1. The Four Molecular Classes

POLEmut tumors carry pathogenic exonuclease domain mutations in DNA polymerase epsilon and behave favorably even where histology looks aggressive. In high-grade endometrioid carcinoma, a meta-analysis of 19 studies and 3092 patients found a pooled prevalence of 11% (95% confidence interval [CI] 9–13). Mutation carried reduced risks of death (hazard ratio [HR] 0.36, 95% CI 0.26–0.50) and progression (HR 0.31, 95% CI 0.17–0.57) [19]. The 2025 European guidelines classify stage I and II POLEmut tumors as low risk, with no adjuvant therapy recommended, and FIGO 2023 assigns them to stage IAmPOLEmut irrespective of grade, LVSI or histotype [9,10]. For stage IIIm POLEmut and IVAm POLEmut disease, the same guidelines state that no firm treatment guidance can be given because data are scarce, but that de-escalation from high-risk treatment can be considered after case-by-case multidisciplinary discussion (level of evidence IV, grade B) [10]. Assignment is not automatic, since only a defined set of exonuclease domain mutations is pathogenic [20].

MMRd tumors arise most often through *MLH1* promoter hypermethylation and carry the immunogenic phenotype supporting checkpoint inhibition. The class is mechanistically mixed, and the mechanism of loss tracks with outcome [21,22].

The p53abn group is defined by aberrant p53 immunohistochemistry as a surrogate for *TP53* mutation. It accounts for 12.2% of 452 population-based tumors [11] and contains most serous carcinomas, but is not confined to non-endometrioid histology. Within high-grade endometrioid tumors, its pooled prevalence is 30% (95% CI 25–34), with worse survival than wild-type p53 (HR 1.29, 95% CI 1.11–1.48) [23].

NSMP is defined by exclusion: no *POLE* mutation, retained mismatch repair proteins, wild-type p53 staining. It is a residual compartment rather than a biological entity, which is the origin of its heterogeneity.

Prognostic separation between the four classes has been reproduced in a population-based validation series of 452 carcinomas [11] and in the TransPORTEC high-risk cohort [24], and partly in an NRG Oncology/GOG cohort of endometrioid carcinomas, in which the copy-number-altered class had significantly worse outcomes and the better outcomes of *POLE*-mutant tumors did not reach significance [25]. In the randomized PORTEC-3 trial, molecular class was strongly prognostic, and chemoradiotherapy significantly improved recurrence-free survival only in p53abn tumors, although the test for interaction between molecular class and treatment was not significant [26]. In the population-based series, class assigned on the preoperative diagnostic specimen matched the hysterectomy result with accuracy 0.91 and κ 0.88 [11]. The information therefore precedes the adjuvant decision.

### 2.2. Residual Risk Within Molecular Classes

**Figure 1:**
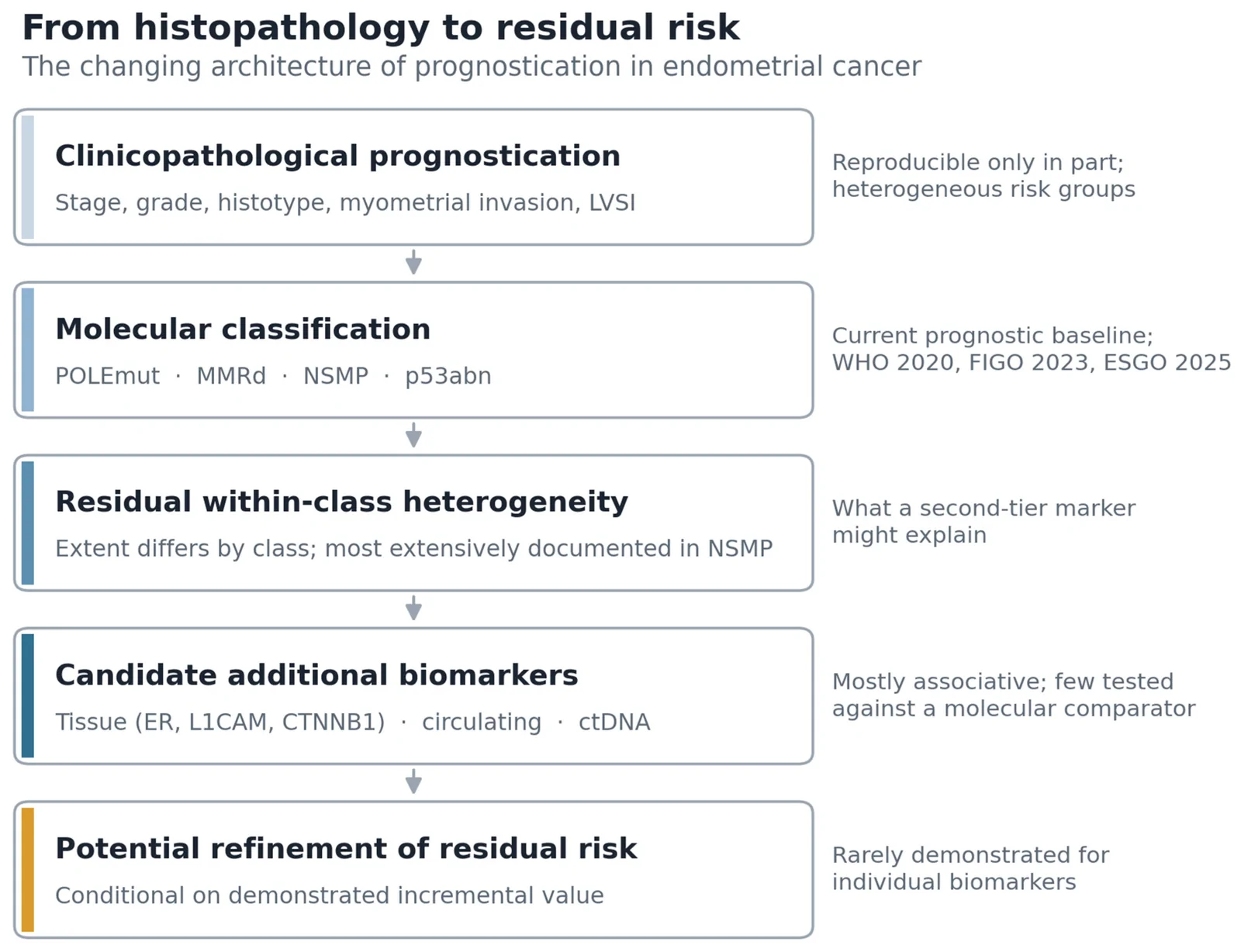
From histopathology to residual risk: the changing architecture of prognostication in endometrial carcinoma. Each layer conditions the one below it rather than replacing it. Clinicopathological variables alone produced risk groups of limited reproducibility. Molecular classification supplied a reproducible baseline now embedded in staging and guidelines. Residual prognostic variation differs in extent and demonstrability across molecular classes and is most extensively documented within NSMP. Candidate tissue, circulating and dynamic markers may explain part of that residual risk. Any refinement of individual risk is conditional on demonstrated incremental predictive performance, so far shown mainly as small gains, in small samples or without external validation. Abbreviations: LVSI, lymphovascular space invasion; POLEmut, *POLE*-ultramutated; MMRd, mismatch repair-deficient; NSMP, no specific molecular profile; p53abn, p53-abnormal; ER, estrogen receptor; ctDNA, circulating tumor DNA.

Molecular class is the starting point of contemporary prognostication rather than its endpoint (Figure 1). Prognostic variation is evident across the classification, although its extent and demonstrability differ by class [25,27]. It is most extensively documented within NSMP. Its pooled overall survival in high-grade endometrioid disease overlaps both POLEmut and p53abn outcomes [12], so the class-level estimate may mask substantial within-group variation; several groups have proposed subclassifying it [28,29].

The other classes are not homogeneous either, though the evidence is thinner and the problems differ. Stage I p53abn low-grade endometrioid carcinoma carries substantial recurrence risk and a hybrid molecular profile, supporting high-risk treatment for p53abn disease with myoinvasion irrespective of grade [30]. Within MMRd disease, the mechanism of repair loss separates outcomes at diagnosis [21,22]. Within POLEmut disease, the difficulty is evidentiary rather than biological: low event rates substantially limit the power to establish second-tier prognostic stratification.

The most instructive demonstration concerns LVSI, a classical variable rather than a novel marker. In 843 patients with stage I–II endometrioid carcinoma, both focal and substantial LVSI predicted worse progression-free survival in MMRd tumors [13]. Only substantial LVSI was prognostic in NSMP tumors, and neither category reached significance in p53abn tumors. These findings persisted in bivariate analyses adjusting separately for stage, grade, estrogen receptor expression, L1CAM, age and adjuvant therapy. The prognostic association of a variable may therefore differ according to the class in which it is measured.

Two consequences follow. Adjuvant decisions based on molecular class alone may not capture residual risk fully, and could contribute to over- or undertreatment in selected patients. The value of any additional marker then depends on where that residual risk lies, currently best characterized within NSMP. The RAINBO program illustrates the transition toward molecularly directed adjuvant treatment [31]. Residual heterogeneity within molecular groups remains a separate problem, requiring refinement that class assignment alone does not supply.

## 3. What Incremental Prognostic Value Means Here

**Figure 2:**
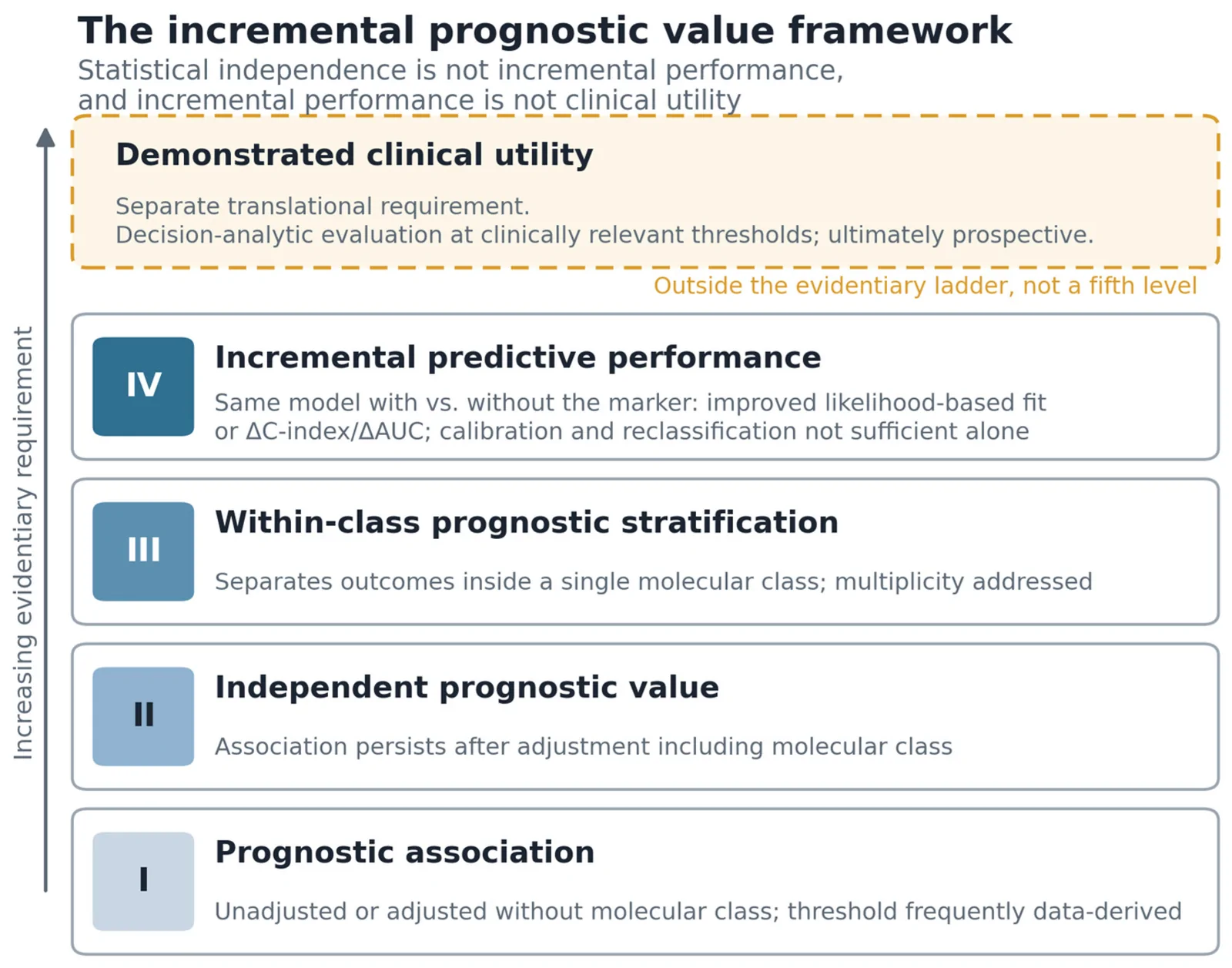
The incremental prognostic value framework. Requirements increase from bottom to top. Level I establishes association, Level II independence after adjustment that includes molecular class, Level III prognostic stratification inside a single molecular class, and Level IV measurable improvement of a comparator model containing molecular class. Evidence at Level IV is further qualified by the comparator used and by whether it was externally validated. An association adjusted for other variables but not for molecular class remains at Level I. At Level IV, calibration and reclassification are reported alongside the change in model fit or discrimination but do not establish the level on their own. Demonstrated clinical utility sits outside the ladder as a separate translational requirement and is not established by statistical performance alone. Abbreviations: ΔC-index, change in concordance index; ΔAUC, change in area under the curve.

The word *prognostic* covers claims of very unequal strength. This review keeps them apart using four levels (Figure 2). The levels are a conceptual framework developed by the authors for this review. Levels I, II and IV follow the distinctions between association, independence after adjustment and improvement in model performance drawn in methodological work on tumor-marker studies and prediction models [14,15], and Level III reflects the within-class question set out in Section 2.2. The framework is not a validated grading instrument, a risk-of-bias tool or a measure of the certainty of evidence, and clinical utility lies outside it rather than forming a fifth level.

Level I. Prognostic association. The marker separates outcomes in unadjusted analysis, or after adjustment that does not include molecular class. Cut-offs are usually chosen in the dataset that tests them, which inflates the effect.

Level II. Independent prognostic value. The effect survives multivariable adjustment that includes molecular class. What counts as adjustment differs widely, and most of the ambiguity in the literature sits here.

Level III. Within-class prognostic stratification. The marker separates outcomes inside a single molecular class. Cohorts shrink sharply, since one class must be analyzed alone. *Discrimination* is reserved here for formal model performance at Level IV.

Level IV. Incremental predictive performance. Adding the marker measurably improves a contemporary comparator model containing molecular class, ideally prespecified and including the relevant clinicopathological variables. Evidence at this level is qualified by the comparator used: improvement beyond molecular classification alone, beyond a full molecular and clinicopathological model, or externally validated. The minimum requirement is a formal comparison, in the same patients, between the comparator containing molecular class and the same model with the marker added, showing a measurable improvement in model fit on likelihood-based comparison or in discrimination, expressed as the change in concordance index (ΔC-index) or in area under the curve (ΔAUC) [15,32]. Calibration should be reported for both models [33], but better calibration alone does not meet the requirement, since miscalibration of the comparator can be corrected without the marker. Reclassification measures can support an improvement shown in this way but do not establish it on their own, because they depend on the risk categories chosen and are easily misinterpreted [34]. External validation is not required to establish that incremental performance was measured; it determines how mature that evidence is. We therefore distinguish internally demonstrated from externally validated incremental performance.

Clinical utility is a separate, further requirement. Improved prediction does not establish that any patient is managed differently or better. Decision-curve analysis brings the question closer to practice, asking whether a model yields more net benefit at thresholds clinicians actually use [35,36]. Net benefit is still a statistical construct, not a demonstrated change in outcome. Each link in the chain has to be established on its own.

Level II causes most of the confusion. A model containing stage and grade was the appropriate comparator before 2013; it is not now. A marker concentrated in one molecular class may appear independent in an unclassified series, because part of what it measures is class membership. What an analysis has shown depends on the comparator, not the *p*-value.

Significance and improvement are different things. A hazard ratio can reach significance while the concordance index barely moves, and reclassification indices can shift without changing any patient’s management [33,34,37]. At Level IV, when the comparator and the extended model are nested and differ by a single parameter, the likelihood-ratio test and the Wald test of the marker’s coefficient, the test usually reported at Level II, can address the same null hypothesis, that the coefficient is zero. They are asymptotically equivalent but not numerically identical, and neither quantifies the gain in discrimination, which is given by the ΔC-index or ΔAUC, nor establishes clinical utility. Calibration deserves separate reporting from discrimination, since a model can rank patients correctly and still assign risks of the wrong magnitude [33].

Two reporting standards cover this ground. REMARK (Reporting Recommendations for Tumor Marker Prognostic Studies) sets out what a tumor-marker study should report about assay, scoring, threshold and covariates [14,38]. TRIPOD (Transparent Reporting of a multivariable prediction model for Individual Prognosis Or Diagnosis) sets out how a prediction model and its validation should be described [39]. Adherence is uneven. Among the studies considered in this review, many of the independent prognostic factors reported therefore cannot be placed above Level II. The marker did not fail. The analysis needed to place it higher was not reported.

Levels were assigned from the analyses reported in the cited studies. Each biomarker was placed at the highest level whose minimum requirement had been met by at least one analysis: for Level I, an association with outcome; for Level II, an association retained in a multivariable model that included molecular class; for Level III, separation of outcomes within one defined molecular class; and for Level IV, the model comparison described above. A higher level was not withheld because a lower one had not been tested, or had not been met in a population spanning several classes, since a signal confined to one class can be diluted across classes, as with estrogen receptor status in high-risk disease (Section 4.1). A level that has not been evaluated is reported as not assessed; this does not constitute evidence against the marker.

Qualifiers specify the setting or comparator to which a level applies. Where a higher level is supported only under restricted conditions, it is reported after the level supported more broadly. Level IV is described as internally demonstrated when the improvement was shown only in the data used to develop the model, with or without resampling-based internal validation, and as externally validated when it was confirmed in patients not used for model development [39]. A range, such as II–III, indicates that, in our judgment, the higher level rests on limited evidence, for example a single cohort, small within-class subgroups or few adjusted analyses; “contested” indicates that analyses at that level reached discordant results. At Level IV, qualifiers are used instead of ranges. A level describes the type of question an analysis has answered, not the certainty of the answer: a Level IV result from one small cohort with internal validation only may be less secure than consistent Level III evidence from several large cohorts, so each assignment should be read together with the limitations given for that biomarker. The design, analysis and reporting standards listed for each level in Table 1 and Figure 2, such as prespecification, control of multiplicity, validation and complete reporting, bear on how securely a level is supported; they are not additional conditions for assigning a level once its minimum requirement has been met.

## 4. Tissue Biomarkers: Refining Risk Within Molecular Classes

### 4.1. Hormone Receptor Status and the Heterogeneity of NSMP

Estrogen receptor (ER) expression is the candidate that has advanced furthest and the one reflected in current European guidance. Receptor loss was an established adverse feature well before molecular classification [40,41]. The question is whether it survives once molecular class is known.

In 648 high-risk carcinomas from the PORTEC-3 trial and a Dutch prospective cohort, none of ER, progesterone receptor (PR), L1CAM or *CTNNB1* was independently prognostic; age, stage and adjuvant chemotherapy were [42]. Subgroup analysis was informative: within NSMP high-risk tumors, ER positivity was independently associated with reduced recurrence risk (HR 0.33, 95% CI 0.15–0.75) [42].

A series of 1110 NSMP carcinomas arrived at the same place from the other direction. Grade 1–2 with ER positivity defined a low-risk subset of 84% of cases, with a five-year disease-specific death rate of 1.6%. The remainder accounted for most deaths (five-year disease-specific death rate 22.9%; HR 16.3, 95% CI 8.4–31.7), independently of stage [43]. An earlier survey had already located most of the hormone-receptor signal in the p53 wild-type compartment [44].

Thresholds matter, and the studies above did not share one. The series of 1110 NSMP carcinomas defined ER positivity as staining in more than 1% of tumor cells, scored on tissue microarrays [43]. The high-risk cohort used 10%, scored on whole slides [42], which is also the threshold specified in the 2025 European algorithm and for eligibility in the NSMP-directed RAINBO trial [10,31]. A multicenter cohort grouped expression as 0–10%, 20–80% or 90–100% rather than using a single cut-off [45]. Three-tiered ER was prognostic within MMRd and NSMP tumors and three-tiered PR within p53-mutant and NSMP tumors; in the p53-mutant subgroup, PR 90–100% corresponded to a five-year disease-specific survival of 100% [45]. Dichotomizing at one arbitrary value discards signal.

The choice of threshold affects which patients are labeled high risk, and can affect what a study appears to show. A tumor with staining between 1% and 10% is receptor-positive under the first definition and receptor-negative under the second, so the two binary rules need not delineate the same subgroup. In the high-risk cohort, a supplementary comparison found the 10% threshold more discriminating than 1% within NSMP, although the authors noted that no consensus threshold exists and that theirs requires validation [42]. The authors of the 1% analysis likewise stated that an optimal cut-off remains to be validated on whole sections [43]. A binary rule also treats every receptor-positive tumor alike. With three tiers, among the 313 NSMP tumors with available ER scoring, 146 (46.6%) fell in the intermediate 20–80% category, and five-year disease-specific survival was 48.0% at 0–10% and 96.0% at 90–100% [45]. Those tiers were taken from an earlier analysis that compared cut-off values for prognostic effect [45]. Three tiers remain a categorization of a continuous measurement, which loses information, and any cut-off chosen in the data on which it is tested overstates the effect [38]. Scoring material, cohort composition and the molecular subgroup analyzed differ as well, and each may contribute to between-study variation. In the three-tiered cohort, the multivariable model reported across all molecular subgroups retained PR category but not ER category [45]; within high-risk NSMP, prognosis followed ER status irrespective of PR status [42]. The cited evidence therefore supports ER as a within-class prognostic marker in NSMP at each threshold used. It does not establish a single optimal cut-off.

The prognostic association of ER beyond NSMP has not been consistently reproduced. A cohort of 899 MMRd carcinomas found that neither ER nor PR, nor grade, histotype, L1CAM, *CTNNB1* or p53 status, added prognostic refinement within that class [22]. Within-class association in one cohort is not the same as a reproducible within-class effect. The ER signal is well supported within NSMP, whereas findings within MMRd remain discordant.

The 2025 update of the European Society of Gynaecological Oncology, European Society for Radiotherapy and Oncology and European Society of Pathology (ESGO–ESTRO–ESP) guidelines uses ER to sub-stratify NSMP disease, defining a subgroup termed NSMP high-grade or estrogen receptor-negative (or both) [10]. In stages describing low-grade endometrioid disease, only ER-negative cases enter it. Among immunohistochemical second-tier markers, ER is the one used this way in the current European framework. Guideline incorporation and incremental predictive performance are nevertheless distinct. The supporting evidence is predominantly Level III. Incremental performance has been tested formally in the high-risk cohort: dividing NSMP by ER status improved the fit and discrimination of a multivariable recurrence model that already contained molecular class (Akaike information criterion 2173.77 versus 2162.38; concordance index 0.712 versus 0.726; *p* < 0.001) [42]. The gain was small, the ER division had been identified in the same cohort, and validation was internal only [42]. That analysis does not show whether the same holds in low- or intermediate-risk disease, at the 1% threshold or with three-tiered scoring. Adoption into guidance is therefore not equivalent to demonstrated incremental predictive performance; in the evidence reviewed here, that demonstration is internal and confined to high-risk disease [42]. Clinical utility remains to be shown: the NSMP-directed RAINBO trial was designed to test de-escalation in ER-positive disease prospectively [31].

An earlier study tested the added-value question directly, before classification was routine. In 293 carcinomas from eleven centers of the European Network for Individualized Treatment of Endometrial Cancer (ENITEC), PR loss was the strongest predictor of recurrence [46]. The three markers combined performed only slightly better than histological variables alone, and the best model contained stage, PR and LVSI. The analysis was appropriate for the comparator standard available at the time, but it does not address the contemporary molecular baseline.

### 4.2. Markers of Aggressive Tumor Biology

L1CAM is a neural cell adhesion molecule whose aberrant epithelial expression tracks with loss of hormone receptors and of E-cadherin [47]. In unclassified cohorts, its prognostic signal is among the strongest reported for any endometrial cancer tissue marker [48,49,50,51]. Expression is strongly histology-dependent: 10% of 935 stage I endometrioid, 18% of 160 advanced endometrioid and 75% of 104 non-endometrioid carcinomas [52].

Does L1CAM identify residual risk after classification? Part of the historical signal is classification itself. In the population-based series of 452 tumors used to validate the classifier [11], L1CAM was expressed in 80% of p53abn cases [53]. A pooled analysis of 849 patients across 13 cohorts put prevalence at 4.87% (95% CI 3.36–6.59) in POLEmut tumors [54]. A marker distributed that unevenly across classes may appear prognostic in an unclassified series because part of its signal reflects molecular-class membership.

Within-class results are more interesting. In that same cohort, L1CAM identified worse outcomes inside the p53 wild-type/NSMP subgroup (*p* < 0.0001) [53]. An Italian series of 94 high-risk tumors reported a prognostic association for L1CAM within NSMP [55]. Korean and Norwegian cohorts reported further stratification alongside the Proactive Molecular Risk Classifier for Endometrial Cancer (ProMisE) [56,57], while a pooled effect on disease-specific survival within MMRd (HR 1.75, 95% CI 1.06–2.89) [54] was not reproduced in the 899-patient cohort [22].

The high-risk cohort analysis found no independent value for L1CAM once molecular class and clinicopathological variables entered the model [42]. In a ProMisE-classified cohort analyzed by risk group, L1CAM expression was associated with adverse clinicopathological features but did not reach statistical significance as an independent prognostic factor; the risk-group-dependent association reported in that study concerned β-catenin (Section 4.3) [58]. L1CAM was not adopted in the 2025 European update [10], although the 2025 meta-analysis highlighted it, together with ER loss, as a potential prognostic marker within NSMP [17].

Why do these results diverge? Several explanations are plausible, and they are not mutually exclusive. One is molecular-class composition. In a ProMisE-classified series, more than 70% of L1CAM-overexpressing tumors were p53abn, and the association with outcome did not persist once molecular subtype entered the model [44]; in the high-risk cohort, L1CAM did not separate outcomes further within p53abn disease [42]. Case mix differs too. The strongest early estimates came from stage I cohorts analyzed before molecular classification [48,49]. In the PORTEC-1 and -2 trials, one of these populations, L1CAM expression above 10%, remained associated with overall survival in high-intermediate-risk disease after adjustment for molecular subgroup, clinicopathological factors and adjuvant treatment (HR 2.46, 95% CI 1.45–4.17) [59]. In a multicenter series, L1CAM was associated with survival in endometrioid but not in non-endometrioid carcinomas, and the authors noted that combining the two in a single high-risk group may have influenced earlier results [52]. The discordant analyses concerned high-risk or mixed-risk populations [42,58]. The covariates modeled also differ. Within high-risk NSMP, L1CAM was not an independent predictor, which the authors attributed to its association with ER and PR negativity [42], in keeping with the link between L1CAM up-regulation and hormone-receptor loss [47]. A Norwegian high-risk cohort differed in three respects: more than half of the tumors were non-endometrioid, most patients received chemotherapy alone rather than radiotherapy-based treatment, and hormone receptors were not modeled. It found the opposite: L1CAM remained significant and molecular class did not [57]. Whether L1CAM appears independent may therefore depend in part on whether ER is in the model.

Precision is a further constraint. NSMP accounted for 21% of 94 tumors in the Italian series [55], and the p53 wild-type subgroup of the Korean series comprised 72 patients, with a 95% CI for the hazard ratio of 1.69–14.29 [56]. These are subgroup analyses, which call for cautious interpretation [38]. Endpoints ranged from recurrence-free [42] and progression-free survival [56] to disease-specific survival [53] and time to recurrence [57]. Assay heterogeneity is a further possible contributor. The positivity threshold was the same, 10% of tumor cells, in several immunohistochemical series cited here [42,46,49,52,57], including two that reached opposite conclusions in high-risk disease [42,57], so the threshold alone does not explain that discordance. Tissue source and platform varied more, from whole sections [42,53] to tissue microarrays [44] and transcript abundance dichotomized at the median [50], which limits comparability with immunohistochemical series [52]; differences in antibody clone or staining protocol cannot be excluded. Pooled hazard ratios within NSMP remain large, at 3.34 (95% CI 2.05–5.44) for progression-free survival [17] and 5.41 (2.92–10.03) in a second meta-analysis [54], but they derive largely from retrospective studies and inherit these limitations.

### 4.3. Genomic Modifiers of Residual Risk

*CTNNB1* exon 3 mutation activates WNT signaling and has been reported in low-grade, early-stage endometrioid tumors [60,61]. In a meta-analysis of seven studies and 1031 patients, the mutation was associated with recurrence in four studies, with 149 patients, that could be pooled for this outcome (odds ratio 3.00, *p* = 0.019) [62]. The pooled disease-free survival hazard ratio was not significant (HR 1.87, *p* = 0.303). Restricted to NSMP and unclassified tumors, that hazard ratio became significant (HR 2.83, *p* = 0.026). This pattern is compatible with a signal concentrated in NSMP, although no test of interaction with molecular class was reported.

Three caveats apply. The included studies are retrospective and contain few mutated cases. The high-risk cohort analysis found no independent effect [42]. The discrepancy may reflect differences in case mix, disease-risk distribution, statistical power or analytical context. And the 2025 meta-analysis found no consistent prognostic significance for *CTNNB1* mutation [17].

The discrepancy with high-risk disease may reflect a difference between settings rather than a contradiction. The positive evidence comes from low-grade, early-stage endometrioid disease. In grade 1–2, stage I–II tumors, mutation was associated with shorter recurrence-free survival on multivariable analysis (HR 5.97, 95% CI 2.69–13.21) [60], and the meta-analysis was confined to FIGO stage I–II endometrioid carcinoma [62]. Even there, the evidence is thin: three of the seven studies contributed to the pooled hazard ratio, and the subgroup estimate was obtained after excluding tumors known to belong to other molecular classes, with molecular status available for only 546 of 886 patients [62]. A molecularly adjusted analysis comes from the PORTEC-1 and -2 trials and was among the studies included in that meta-analysis [62]. Among 546 women with early-stage, high-intermediate-risk endometrioid carcinoma, mutation status was known in 534 and was not associated with distant recurrence in univariable analysis (HR 0.93, 95% CI 0.45–1.93). After exclusion of p53-mutant and *POLE*-mutant tumors and of tumors with substantial LVSI or L1CAM expression above 10%, it was associated with distant recurrence in a multivariable model that included molecular subgroup, microsatellite-unstable or NSMP (HR 2.96, 95% CI 1.23–7.10; 443 women, 23 events) [59]. Covariates were selected and estimates obtained in the same data, and microsatellite-unstable and NSMP tumors were analyzed together, so the result is a molecularly adjusted association in a selected early-stage subgroup, not evidence of stratification within NSMP. The negative evidence comes from high-risk disease, and the authors of that analysis suggested that the larger share of low- and intermediate-risk patients in earlier studies might explain the difference [42]. Because mutation clusters in low-grade tumors with lower rates of deep myometrial invasion and lymphovascular space invasion [60], a high-risk cohort contains proportionally fewer of the patients in whom the signal was described. That could contribute to the absence of an effect, although it has not been tested directly. Marker definition differs as well. Some series identified mutation by sequencing [42,60]; others used β-catenin immunohistochemistry [56,58], for which nuclear staining had a reported sensitivity of 91% and specificity of 89% for exon 3 mutation [61] and which can miss focal staining [62]. With immunohistochemistry, β-catenin was not associated with recurrence in one ProMisE-classified cohort [56] and was associated with progression-free survival only in the high-intermediate-risk subgroup of another [58]. Co-occurring alterations may modify the effect, as the *ARID1A* data below suggest [63]. A further meta-analysis, restricted to NSMP disease, found no significant effect of *CTNNB1* mutation on recurrence or survival [16].

*ARID1A* sits in a similar position. In 125 profiled carcinomas, NSMP tumors carrying *ARID1A* mutation had the worst outcome, while those with wild-type *ARID1A* and altered β-catenin recurred in no case [63]. The 2025 meta-analysis found an unfavorable effect confined to NSMP [17]. The supporting studies are few and none has been reproduced in a trial cohort. Phosphoinositide 3-kinase (PI3K)-pathway alterations, taken as a category, reach Level I, contested. In the series of 1110 NSMP carcinomas, *PIK3CA* mutation was associated with disease-specific survival on univariable analysis [43]; this places *PIK3CA* mutation, but not the pathway as a whole, at Level III, contested, since *PIK3CA* and *PTEN* mutations showed no consistent prognostic significance, overall or within NSMP, in the 2025 meta-analysis [17].

Copy-number alterations offer a further candidate. Amplification of chromosome 1q32.1, a region that includes *MDM4*, was associated with shorter recurrence-free survival within the NSMP subgroup of a discovery series pooling 141 array-profiled and 361 TCGA tumors, after adjustment for age, stage and grade (HR 2.12, 95% CI 1.26–3.59), and the association was replicated in 487 NSMP tumors from the PORTEC-1 and -2 trials after adjustment for age, grade, LVSI and adjuvant treatment (recurrence-free survival, HR 2.34, 95% CI 1.17–4.70; distant recurrence, HR 3.98, 95% CI 1.72–9.17); no significant association was found in microsatellite-unstable or *TP53*-mutant tumors [64]. This is Level III evidence, replicated in an independent cohort. The two cohorts differed, however, in setting, from all stages in the discovery series to early-stage trial disease, and in assay, from genome-wide single-nucleotide polymorphism arrays to targeted multiplex ligation-dependent probe amplification, and the amplified category combined focal 1q32.1 events with amplification of the whole 1q arm. The reported improvement in risk prediction rested on survival differences between the groups of a five-group classification rather than on a comparison of model performance, and clinical utility has not been evaluated [64]. In an exploratory analysis of an NSMP series profiled by targeted sequencing, a genomic cluster characterized partly by high-level 1q gain was associated with worse overall survival, but 1q copy-number status alone was not prognostic on multivariable analysis [65]. Assay, copy-number definition and case mix differed from those of the 1q32.1 studies, so the two results are not directly comparable.

### 4.4. What Survives Adjustment

Markers that looked strongly prognostic before classification often lose independence afterwards, because part of what they measured was class membership; L1CAM in p53abn disease is the clearest case. Several of those that survive do so inside particular classes, with ER within NSMP best supported, and LVSI shows that even an established variable can behave differently across classes [13].

Among the studies identified, formal analyses of incremental predictive performance are uncommon, and for conventional second-tier tumor markers the gains they show are small, rest on very few patients or come from a model without external validation. Supplementary Table S2 summarizes these analyses, with the comparator, cohort size, number of events where reported, and validation of each. In one molecularly characterized cohort, adding ER and L1CAM status, peritoneal washing status and tumor diameter above 5 cm to a Cox model of clinicopathological variables fitted within the p53abn class raised the concordance index for disease-specific survival from 0.764 to 0.774 [66]. A tree-based model gained more, from 0.725 to 0.794 in the 87 p53abn patients and from 0.789 to 0.884 in 305 MMRd patients [66]. Because the four variables were added together, these gains cannot be attributed to ER or L1CAM individually. None of these results were externally validated. The ER analysis described in Section 4.1 showed a small gain, similar to that of the Cox model, from 0.712 to 0.726, again without external validation [42]. For L1CAM on its own, one such comparison was identified. Models developed in 94 high-risk patients were applied, after recalibration, to an external cohort of 47; within its 22 NSMP patients, of whom 18% relapsed or died of disease, the concordance index for progression-free survival was 0.75 with L1CAM and 0.68 without it [55]. No confidence interval or test of the difference was reported, and no such comparison was reported for the development cohort. With so few events in a cohort the authors described as small [55], this imprecise difference is limited evidence from one externally tested comparison, not validation of an established incremental effect. In 899 MMRd carcinomas, grade, histotype, ER, PR, L1CAM, *CTNNB1* and p53 status added no prognostic refinement, whereas the mechanism of mismatch repair loss did [22].

One of the few direct assessments of incremental performance against a molecular comparator comes from genomic instability rather than from a conventional marker. In 142 patients from a single center, homologous recombination deficiency (HRD) subtyping based on genomic scar patterns was compared with, and added to, TCGA classification [67]. Concordance indices for overall survival were 0.751 for TCGA classification alone, 0.857 for HRD subtyping alone and 0.903 for the two combined; the corresponding values for disease-free survival were 0.669, 0.787 and 0.848 [67]. This directly tests incremental performance beyond molecular classification, and its limits are instructive. Subtypes were derived by unsupervised clustering and the threshold by maximally selected rank statistics, both in the cohort that then tested them. Only 18 patients relapsed and the HRD-high cluster contained 12 patients. There was no external validation. The comparator was TCGA classification alone rather than a full molecular and clinicopathological model. The result therefore sits at Level IV as internally demonstrated incremental performance beyond molecular class, and the magnitude should be read as optimistic.

A gain has also been reported for integrated multimodal prediction rather than for any single marker. A deep learning model using whole-slide images and stage was trained and tested in 2072 patients from eight cohorts, including the PORTEC trials, of whom 1408 formed the training set. In cross-validation within the development data, its concordance index for distant recurrence was 0.795, against 0.762 for a model combining histological type, grade, LVSI, stage and molecular class, and the authors described its discrimination as better than or as good as that of this model [68]. In held-out test sets it reached 0.789 internally and 0.828 and 0.815 in two external cohorts [68]. Because the deep learning model replaces the comparator rather than adding a marker to it, this is not the comparison of the same model with and without a marker that Level IV requires, and the gain over the comparator given above was obtained by cross-validation in the development data. What that study establishes is discrimination in held-out cohorts. It reports no decision-analytic evaluation, and clinical utility remains to be shown prospectively.

Where such analyses have been done, the gains have been modest, with increases in the concordance index of about 0.01 to 0.03, whether a conventional second-tier marker [42,66], immune contexture [69] or multimodal integration [68] was assessed. Larger increases came from small samples, namely 22 patients for L1CAM [55] and 142 patients with 18 relapses for genome-wide instability subtypes derived in the same cohort [67], or from a flexible tree-based model without external validation [66]. For most of the individual markers examined here, incremental predictive performance has not been evaluated at all (Table 2), which is not evidence that it is absent.

## 5. Circulating Biomarkers: Independent Information or Surrogates of Tumor Burden?

### 5.1. CA125 and HE4

Elevated preoperative cancer antigen 125 (CA125) tracks with advanced stage, nodal involvement and shorter survival, and a meta-analysis of pretreatment values confirmed the association [70,71].

One study examined the question within molecular classes. In a ProMisE-classified cohort, the proportion of patients with CA125 above 35 kU/L was lowest in POLEmut and highest in p53abn disease [72]. Abnormal CA125 was associated with higher stage and lymph node metastasis in the cohort as a whole and within each subtype, on univariable and multivariable analysis [72]. Elevated CA125 was also associated with poorer survival within the p53abn, NSMP and MMRd molecular subgroups [72]. Its accuracy for identifying nodal metastasis was limited [72]. CA125 therefore provides risk enrichment rather than a stand-alone rule-in or rule-out test.

Whether this is prognostic information distinct from tumor burden remains unresolved. Both markers rise with the anatomical extent that staging already captures, and FIGO 2023 captures it more precisely through peritoneal carcinomatosis and refined nodal categories [9]. A marker that predominantly reflects tumor burden may add less once anatomical extent is characterized more precisely, whereas a biologically orthogonal marker may retain value. Among the studies identified here, one multivariable model retained CA125 alongside stage and molecular class (Section 5.2), which is not sufficient to show that either CA125 or human epididymis protein 4 (HE4) behaves in the second way once molecular class is known.

Preoperative measurement has a separate and more defensible role. CA125 improved preoperative risk stratification in high-grade disease [73] and in a prospective low-grade cohort [74], and HE4, alone or with CA125, has been used to predict nodal metastasis and monitor recurrence [75,76,77]; preoperative CA125 has also been associated with nodal metastasis [78]. This is triage before surgery, not refinement of postoperative risk.

### 5.2. Systemic Inflammatory Indices

The neutrophil-to-lymphocyte ratio (NLR), platelet-to-lymphocyte ratio (PLR) and related indices come from routine blood counts at no extra cost. A meta-analysis of nine studies and 3390 patients examined pretreatment values [79]. Elevated NLR was associated with shorter overall survival (pooled HR 2.22, 95% CI 1.77–2.78) and worse disease-free survival (1.81, 1.35–2.41). Elevated PLR gave comparable estimates for overall survival (1.99, 1.51–2.61) [79]. A later meta-analysis of 14 studies confirmed the associations for NLR and PLR but found none for the monocyte-to-lymphocyte ratio [80].

Effects of that size would matter if the evidence were homogeneous. Cut-offs are usually derived in the dataset that tests them, endpoints and adjustment sets differ, and biological specificity is low: these indices respond to infection, obesity, thromboembolism and cardiovascular disease, all common here. Neither pooled analysis adjusted for molecular class, because the constituent studies predate routine classification.

One multicenter study combined an inflammatory-nutritional index with molecular class. The study comprised 582 patients (365 in a training cohort and 217 in a separate external validation cohort); its multivariable model for recurrence-free survival contained age, stage, grade, histological type, LVSI, myometrial invasion, CA125, ProMisE subgroup, the hemoglobin–albumin–lymphocyte–platelet score and adjuvant therapy [81]. Age, grade, LVSI, CA125, the score and adjuvant therapy were significantly associated with recurrence-free survival. For stage and ProMisE subgroup, which the authors also listed as independent predictors, the global tests were not significant (*p* = 0.113 and *p* = 0.067), although stage III versus stage I and p53abn versus p53 wild-type tumors had significant coefficients (*p* = 0.042 and *p* = 0.030) [81]. The resulting nomogram was evaluated in both the training and the external validation cohort by concordance index, calibration and decision-curve analysis. This study addresses the relevant modeling question, although limitations in model development and reporting warrant caution when interpreting the findings. What remains untested is the incremental contribution of the score itself, which is what would place it at Level IV: the complete model was not compared with an otherwise identical model omitting the score.

### 5.3. What Blood-Based Markers Have and Have Not Shown

CA125 has now been characterized within molecular subtypes, which puts it ahead of the rest. Among the circulating markers examined here, none has been shown to improve a model that already contains molecular class. The inflammatory indices remain at Level I, with one exception: the hemoglobin–albumin–lymphocyte–platelet score retained independent prognostic value in a model that included the ProMisE subgroup, which places it at Level II [81]. This applies to the indices analyzed in the studies cited; the systemic immune-inflammation index and indices based on C-reactive protein were not analyzed there as individual markers, so no level is assigned to them. Until thresholds are prespecified and comparator models contain molecular class, this literature will keep accumulating associations. Table 3 summarizes the evidence level, clinical relevance and main limitations of the circulating biomarkers considered in this review.

## 6. Emerging Biomarkers and the Next Layer of Prognostication

### 6.1. Circulating Tumor DNA

Circulating tumor DNA (ctDNA) introduces a different kind of information. Tissue markers describe tumor biology as it existed at resection. Detectable ctDNA after surgery describes residual disease, which is a different quantity and is measured repeatedly rather than once. It should therefore not be read as one more second-tier tissue marker.

Across resectable solid tumors, ctDNA-based detection of molecular residual disease is strongly associated with recurrence, and pooled evidence supports both landmark and longitudinal testing [82]. None of the 80 studies pooled concerned endometrial cancer, and sensitivity for recurrence was 0.50 with a single landmark test against 0.74 with longitudinal testing, at a specificity of about 0.90 for both [82]. Endometrial cancer-specific data are younger. Postoperative tracking of tumor-derived mutations, but not detection before surgery, was associated with relapse in high-risk disease [83]; high-sensitivity assays have been applied to monitoring [84]; post-surgical residual disease has been assessed in stage I uterine malignancies [85]; and preoperative and serial sampling has been reported in a prospective multicenter cohort [86]. Cohorts remain small, at 9, 44, 101 and 198 patients [83,84,85,86]. Designs range from prospectively sampled cohorts [84,86] to a real-world analysis with a median of 6.8 months of ctDNA monitoring [85], and several high-sensitivity approaches are tumor-informed and resource-intensive.

The timing of sampling matters. Before surgery, detection is associated with advanced stage and other high-risk features [84,86], so a preoperative result partly reflects disease extent that staging already captures. It is the postoperative result that corresponds to molecular residual disease. Adjustment for molecular class has been partial. Postoperative ctDNA remained associated with recurrence after adjustment for histological risk group, mismatch repair and p53 status [85]. A preoperative combination of total cell-free DNA and ctDNA was reported to be independent of clinicopathological factors and molecular subtype, whereas preoperative ctDNA alone was not independent of clinical variables [86]. None of the endometrial cancer studies identified here has formally demonstrated incremental predictive performance over a contemporary four-class molecular model. None evaluated ctDNA-guided management.

Two features separate ctDNA from everything discussed so far. It is dynamic, so it can monitor rather than only predict at baseline. And it is plausibly complementary to molecular class rather than competitive with it, since class describes biology while ctDNA describes persistence of disease. Prospective evaluation inside molecularly stratified trial populations is the appropriate next step [31,87]. The steps this would involve are set out in Section 6.4.

Because of these features, ctDNA is discussed separately and is not assigned a level within the framework applied in Table 2 and Table 3. Those levels were framed for a characteristic measured once and judged against molecular class determined at the same time. A ctDNA result means different things at different times: before surgery it may add to baseline stratification, after surgery it indicates molecular residual disease, and in serial samples it is used for monitoring. Prognostic association can be examined at each of these points. Added predictive performance is most clearly defined for a single landmark result, judged against a comparator fixed at the same time point, such as a postoperative model containing molecular class and stage, whereas monitoring is judged by sensitivity and specificity for recurrence [82] and by lead time over clinical detection [86]. Management guided by ctDNA is a further question of clinical utility, which only a comparison of management strategies can answer.

### 6.2. Immune and Microenvironmental Biomarkers

Immune infiltration has to be interpreted conditionally on molecular class, since POLEmut and MMRd tumors carry high neoantigen loads and dense infiltrates, so any unadjusted analysis will partly recapitulate classification. Programmed death-ligand 1 (PD-L1) illustrates the problem: pooled across cohorts with associations to stage and lymphovascular invasion, but without consistent molecular adjustment [88].

Two analyses answer different questions and support different levels. In 695 stage I endometrioid tumors from PORTEC-1 and PORTEC-2, intraepithelial CD8-positive density was selected from fifteen marker-compartment combinations on effect size and Akaike information criterion (unadjusted HR 0.88 per two-fold increase, 95% CI 0.82–0.95) [69]. In the 640 patients with complete covariate data analyzed in the multivariable models, the association with recurrence persisted after adjustment that included molecular class (adjusted HR 0.88, 95% CI 0.81–0.95) [69]. Comparison of multivariable models, again with internal bootstrap validation, showed that the molecular-immune model had the highest concordance (0.726 for recurrence, against 0.697 for the same model without CD8 density), and CD8 density outweighed myometrial invasion, grade and L1CAM as a predictor of recurrence [69]. That is a measured improvement on a model already containing molecular class, so it meets the Level IV definition as internally demonstrated. No external validation, calibration or decision-analytic evaluation was reported, and clinical utility is not established.

The second analysis works inside a single class. In 899 MMRd carcinomas, lower CD8 density identified worse outcomes and *MLH1* loss was associated with lower density, which is Level III [22]. The two do not agree on where the immune signal lies: an exploratory analysis of the PORTEC trials suggested that the association was greatest in p53-mutant tumors and absent in MMRd disease [69]. Immune contexture is therefore one of the few domains reviewed here in which incremental predictive performance has been measured, although its distribution across molecular classes remains contested.

### 6.3. Multi-Parameter Signatures

Transcriptomic, proteomic and integrated signatures extend the same logic, and the methodological requirements rise accordingly. A signature developed and evaluated in the same cohort is at substantial risk of optimistic performance estimates. Performance in an independent external dataset, not optimism-corrected internal performance, provides the more stringent test [39]. Among the approaches reviewed here, computational pathology carries some of the strongest external-validation evidence. One interpretable model predicts molecular class from routine slides, with the PORTEC-3 trial held out as a test set [89]. The multimodal model discussed in Section 4.4 is the other [68].

A practical constraint applies to all of them. Any signature meant to refine molecular class must run on the same formalin-fixed material within the timeframe of an adjuvant decision. Assays requiring fresh tissue, prolonged turnaround or specialized infrastructure are less likely to reach routine use. That is one reason ER, a single inexpensive stain, advanced faster than any genomic modifier.

### 6.4. Future Perspectives: From Emerging Biomarker to Routine Use

The markers in this section share a prognostic association and differ in how far they have traveled beyond it. Routine use requires a sequence of demonstrations, each of which has to be established on its own: a reproducible assay, prognostic validity in molecularly classified cohorts, incremental predictive performance against a prespecified contemporary comparator, external validation, and evidence that acting on the result improves decisions or outcomes [14,15,35,39]. Multimodal computational pathology has reached external validation of discrimination [68], and intraepithelial CD8-positive density an internally demonstrated increment [69]. In endometrial cancer, ctDNA has not yet completed the first two.

For ctDNA, the requirements are specific, because the quantity measured is residual disease over time rather than a fixed property of the tumor. The assay comes first. Patient-specific assays track mutations identified in each patient’s tumor [83,85,86], whereas one study sequenced plasma with a fixed 129-gene panel and called variants against the matched tumor [84]; the two approaches trade sensitivity against breadth of coverage [86]. Pooled data from other tumor types show that detection technology influences sensitivity, and that neither the definition of a positive result nor panel content and sequencing depth are standardized [82]. Limits of detection, reproducibility between laboratories and the threshold for positivity therefore need to be fixed before clinical comparison is meaningful [14,38].

Clinical validation then requires prospective cohorts in which molecular class is known and sampling times are specified in advance, with both a postoperative landmark sample and serial samples. The distinction is not a technicality. A single landmark test missed about half of recurrences in the pooled data, so a negative result cannot by itself support de-escalation, whereas serial testing raised sensitivity at similar specificity [82]. In the largest endometrial cohort cited here, sampling was mainly six-monthly, a sample one month after surgery was taken in only 37 patients, and ctDNA was not detected in four of the 22 patients whose disease progressed [86]. Enough events are needed within each molecular class, which will probably require multicenter collaboration or sampling embedded in molecularly stratified trials [31,87].

The third step is the one this review has asked of every marker. Postoperative ctDNA status should be added to a prespecified model containing molecular class, stage and the established clinicopathological variables, with the change in discrimination and calibration reported and then confirmed in an independent cohort [15,33,39]. Because ctDNA measures something that molecular class does not, the gain could be substantial. That remains a hypothesis: no endometrial study identified here reported the change in discrimination or calibration when ctDNA was added to such a model. The last step is clinical utility. Detection ahead of clinical recurrence has been reported [84,86], by a mean of 4.7 months in the larger of the two cohorts [86], but earlier detection is not a benefit in itself unless an effective intervention exists and is shown to change outcome. Demonstrating utility requires actions defined in advance for a positive and for a negative result, decision-analytic evaluation at those thresholds [35,36], and ultimately a controlled comparison of ctDNA-guided standard management, ideally randomized. A comparison of this kind has been called for across tumor types [82]. Until then, the position can be stated plainly. In the endometrial cancer studies reviewed here, ctDNA has shown prognostic association. It has not been shown to add incremental predictive performance to a contemporary molecular and clinicopathological model, and its clinical utility has not been demonstrated. On current evidence, it is not ready for routine use.

The same sequence applies to the other emerging markers, with different bottlenecks. For intraepithelial CD8-positive density, the missing steps are a quantification method reproducible in routine pathology, external validation and calibration [69]. For multimodal computational models, discrimination has been validated externally [68]; prospective and decision-analytic evaluation has not been reported.

## 7. From Additional Biomarkers to Better Prognostic Models

The structure that emerges is layered rather than additive: molecular class, clinicopathological context, a within-class modifier once validated, then a dynamic marker of residual disease.

PORTEC-4a tested a version of the first three layers prospectively, as a strategy for allocating adjuvant treatment. Women with high-intermediate risk endometrial cancer were randomized in a 2:1 ratio to adjuvant treatment guided by a molecular-integrated risk profile or to standard vaginal brachytherapy, and the final analysis comprised 564 women, including 23 with a favorable profile added from the PORTEC-4 trial [90]. The profile, developed from analyses of the PORTEC-1 and -2 trials [59], combined molecular class with LVSI, *CTNNB1* mutation status and L1CAM expression. A favorable profile received no adjuvant treatment, an intermediate profile received brachytherapy, and an unfavorable profile received external beam radiotherapy [90,91]. Median follow-up was 58.1 months. The five-year cumulative incidence of vaginal recurrence was 4.5% (95% CI 2.23–6.76) in the molecular-profile arm and 1.6% (0.00–3.32) in the standard vaginal brachytherapy arm (hazard ratio 2.71, 95% CI 0.79–9.34). Non-inferiority was assessed on the absolute difference in this incidence: the upper bound of the one-sided confidence interval of the difference, 5.3 percentage points, was below the predefined margin of 7.0 percentage points (*p* = 0.005). In the molecular-profile arm, 46% of women had a favorable profile and received no adjuvant treatment [90].

The trial therefore evaluated a molecular-integrated treatment strategy, and its result supports that strategy as a whole. Because of the allocation, fewer women in the molecular-profile arm received adjuvant treatment, and adverse events did not differ substantially between the arms [90]. The result does not show the independent prognostic contribution or the incremental predictive performance of L1CAM or of *CTNNB1*, which served within the profile to allocate treatment. The profile was tested as a package against a single-treatment comparator, not against the same profile with those markers removed. Reading PORTEC-4a as validation of a specific marker asks more of the design than it can support. Workflow feasibility had been shown in the pilot phase [92], and further molecular-guided programs are running [31,87].

Several limitations recur among the studies considered in this review. Most evidence comes from retrospective cohorts assembled before classification became routine, class-specific subgroups are small, multiplicity is rarely addressed and endpoints vary. Cut-offs are heterogeneous and frequently data-derived, and the ER literature shows how threshold choice can change what a study appears to show, while scoring material and case mix may also contribute [42,43,45]. Adjustment for molecular class is often absent, external validation uncommon, and calibration infrequently reported.

Differences between the source studies were handled descriptively. No estimates were pooled, so heterogeneity was not assessed statistically; each result is instead reported with the population, methods and comparator that qualify it, and discordant findings are discussed where such differences might explain them. Among the analyses of incremental performance, molecular class was assigned by immunohistochemistry combined with *POLE* sequencing in three [42,55,69], by ProMisE with imputation of missing classes in one [66] and by a sequencing-based surrogate in another [67]; the multimodal model used classes predicted from routine slides, and missing classes were imputed for its comparator [68]. Assays and thresholds varied, as discussed for ER (Section 4.1) and L1CAM (Section 4.2). Populations ranged from stage I endometrioid disease in trial cohorts [69] to high-risk [42,55] and unselected series [66], and the proportion of non-endometrioid tumors differed [52,57]. Treatment differed with era and setting, from radiotherapy-based regimens in a cohort treated from 1987 onwards [42] to chemotherapy alone in most patients of another high-risk cohort [57]. Endpoints, length of follow-up and, above all, the comparator model also differed (Supplementary Table S2).

Small numbers were handled in the same way. Sample sizes and event numbers are given with the principal results, and the qualifiers defined in Section 3 indicate where a level rests on limited evidence. Several results that shape the conclusions rest on small numbers: an HRD-high cluster of 12 patients in a cohort with 18 relapses [67], 22 NSMP patients in an external cohort [55], 87 p53abn patients [66], a p53 wild-type subgroup of 72 patients [56], and 62 p53abn patients and a single progression among POLEmut tumors in the LVSI analysis [13]. Event numbers were not available for every analysis (Supplementary Table S2). Within-class findings are subgroup analyses by definition, which call for cautious interpretation [38]; in the CD8 study, the authors described the class-specific differences in the prognostic value of CD8 density as exploratory findings requiring validation [69]. Multiplicity also arises in marker selection: intraepithelial CD8 density was chosen from fifteen marker-compartment combinations in the cohort that then tested it [69]. This review did not re-analyze data or correct for multiple testing. It reports these features so that the weight of each finding can be judged, and it attributes limitations stated by the investigators to them, separately from those identified here.

### Limitations of This Review

Several limitations qualify the conclusions above. Coverage was purposeful rather than exhaustive, so selection bias cannot be excluded. Such selection may favor markers that have been studied extensively or are clinically prominent, and may under-represent less developed candidates and negative findings that remained unpublished. We mitigated this by presenting discordant results alongside supportive ones, particularly for L1CAM, *CTNNB1* and ER outside NSMP, and by citing the two recent meta-analyses whose emphases differ [16,17]. Exhaustive coverage was not attempted because the review asks what kind of evidence exists for markers that have reached molecularly annotated study, not what their pooled effect is; markers without such data are absent by design rather than judged unimportant. Level assignment also involved judgment. It was made by the authors from the published analyses, without independent duplicate assignment or assessment of inter-rater agreement; the explicit rules in Section 3 allow each assignment to be checked against the cited analyses, which is not the same as inter-rater validation. The field is also moving quickly. The 2025 European guideline update and the first randomized results of molecular profile-guided treatment both appeared while this review was being prepared, so the conclusions drawn here are provisional and may be superseded as further evidence accrues.

## 8. Where the Evidence Still Falls Short

Four observations define the current position.

Much of the biomarker literature predates routine molecular classification. Studies reporting independence after adjustment for stage and grade were correct by the standards of their time but are no longer sufficient, because the comparator has changed.

Multivariable significance is often read as incremental value in the studies considered here. A significant hazard ratio establishes association conditional on covariates. It says nothing about whether adding the marker improves discrimination, calibration or risk-group assignment [33,34].

Evidence is distributed unevenly across the four classes, and the unevenness is structured rather than accidental. It is dense within NSMP, a category defined by exclusion in which residual variation is most extensively documented. Within MMRd, it is discordant. Conventional immunohistochemical and genomic markers did not demonstrate additional prognostic refinement in a cohort of 899 MMRd tumors, whereas the mechanism of mismatch repair loss and CD8 density were associated with differences in outcome [22]. An exploratory trial-based analysis suggested that the CD8 association was absent in that class [69]. For p53abn disease, part of the signal reported for markers such as L1CAM in unclassified cohorts reflects class membership, since L1CAM is expressed in 80% of these tumors [53]. Within POLEmut, the constraint is evidentiary rather than biological, since favorable outcomes and low event rates substantially limit the power to establish second-tier stratification [13,44].

External validation and decision-analytic evaluation remain uncommon. Among the few analyses that measured incremental predictive performance, gains from conventional second-tier tumor markers were small [42,66]. Gains of the same order were reported for intraepithelial CD8 density added to a molecular model [69] and for multimodal prediction [68]. Larger increases came from small samples, as in the single comparison for L1CAM [55] and for a genome-wide instability metric derived in the cohort that tested it [67], or from a flexible tree-based model without external validation [66]. Apart from the L1CAM comparison in 22 patients [55], only the multimodal model was evaluated in external cohorts, in which its discrimination was maintained [68]. These observations rest on few analyses and should be read as a direction rather than a conclusion.

Future studies should prespecify the comparator model, and it should contain molecular class rather than stage and grade alone. Prespecified thresholds, reporting aligned with REMARK and TRIPOD, and external validation in molecularly annotated cohorts would resolve most of the present ambiguity [14,38,39]. For emerging markers, and for ctDNA in particular, the further steps required before routine use are set out in Section 6.4.

## 9. Conclusions

Molecular classification is the appropriate baseline for prognostic assessment in endometrial carcinoma, although residual prognostic variation differs in extent and demonstrability across molecular classes. Estrogen receptor status has advanced furthest toward routine implementation among the second-tier markers reviewed here, and now sub-stratifies NSMP disease in European guidance. That incorporation rests predominantly on within-class prognostic evidence. It is distinct from demonstrated incremental predictive performance, which for estrogen receptor status has been shown only in high-risk disease and with internal validation, and from clinical utility, which for estrogen receptor-guided management remains to be established. Beyond that, the evidence supports association and, in places, within-class prognostic stratification, but it rarely supports incremental predictive performance and for none of the individual biomarkers examined here is prospective clinical utility established in isolation. These are different claims, and in the literature reviewed here they are frequently collapsed.

A second conclusion concerns where refinement is plausible at all. The evidence is not simply thinner in some classes than others; it is unevenly distributed for four distinct reasons, one per class. NSMP is heterogeneous by construction and yields the most candidates. Within MMRd, evidence for conventional prognostic markers remains inconsistent, whereas the mechanism of mismatch repair loss provides additional prognostic stratification and findings for immune markers remain discordant. For p53abn disease, part of the signal reported in unclassified cohorts is class membership itself, and in POLEmut disease low event rates limit the power to establish second-tier stratification. Progress will come from testing selected markers against that map, with prespecified comparators and external validation, rather than from lengthening the list of markers that are prognostic.

## Figures and Tables

**Table 1:** Design, analysis and reporting standards expected at each level of the evidentiary framework, together with the failure mode most often seen at that level.

Level	Claim (Minimum Requirement) and Design	Expected Analysis and Reporting Standard	Failure Mode Most Often Seen
I. Prognostic association	Marker is associated with outcome. Cohort with biomarker and outcome data	Survival comparison, unadjusted or adjusted without molecular class; effect size with 95% CI. REMARK items on assay, scoring and threshold [14,38]	Cut-off optimized in the analysis dataset
II. Independent prognostic value	Association survives adjustment that includes molecular class. Cohort with relevant covariate information and appropriate handling of missing data	Multivariable model including molecular class and clinicopathological variables, prespecified covariate set, full model reported [14]	Adjustment limited to stage and grade, with molecular class absent from the comparator
III. Within-class prognostic stratification	Marker separates outcomes inside one molecular class. Cohort large enough for class-specific analysis	Analysis restricted to one class, with multiplicity addressed where applicable, prespecified and labeled as a subgroup analysis [14]	Post hoc subgroup presented as a primary result
IV. Incremental predictive performance	Marker improves a comparator containing molecular class, ideally prespecified. Development cohort; comparator quality and external validation determine evidence maturity	Comparison with the same model without the marker, showing improved likelihood-based model fit or discrimination (ΔC-index or ΔAUC) [15,32]; calibration reported for both models and reclassification interpreted cautiously, neither sufficient alone [33,34,37]; reported per TRIPOD [39]	Significance of a coefficient offered in place of model improvement; internal performance reported as if externally established
Beyond Level IV: clinical utility	Using the marker changes decisions for the better	Decision-analytic evaluation at clinically relevant thresholds [35,36]; ultimately prospective evaluation	Improved discrimination presented as demonstrated clinical benefit

Levels are an analytical framework developed for this review and were not used by the original studies. AUC, area under the curve; C-index, concordance index; CI, confidence interval; REMARK, Reporting Recommendations for Tumor Marker Prognostic Studies; TRIPOD, Transparent Reporting of a multivariable prediction model for Individual Prognosis Or Diagnosis.

**Table 2:** Evidence level reached by each candidate tissue biomarker within the framework of this review, together with its clinical relevance and main limitations. In the upper block, columns two to four correspond to Levels I–II, III and IV as defined in Section 3, and the last column gives the level assigned; the lower block gives the clinical relevance and main limitations of each biomarker.

Biomarker (Domain; Class Where the Signal Concentrates)	Association and Adjustment for Molecular Class (Levels I–II)	Within-Class Evidence (Level III)	Incremental Predictive Performance (Level IV)	Evidence Level Within This Review Framework
ER—hormone receptor signaling; NSMP well supported, MMRd discordant	Consistent association [40,41]; not independent in the whole high-risk cohort [42]	HR 0.33 (0.15–0.75) in NSMP high-risk disease [42]; low-risk NSMP subset with grade, 84% of 1110 cases, HR 16.3 (8.4–31.7) for the remainder [43]; three-tiered cut-off prognostic in MMRd and NSMP [45], not reproduced in 899 MMRd tumors [22]	Assessed in high-risk disease: model fit improved when NSMP was divided by ER status (likelihood-based comparison, *p* < 0.001; C-index 0.712 vs. 0.726); internal validation only [42]	III; IV, internally demonstrated in high-risk disease only
PR—hormone receptor signaling; NSMP, p53abn	Consistent association [40,41]; not independent in the whole high-risk cohort [42]; three-tiered PR independently prognostic in a model containing molecular subgroup in one retrospective cohort [45]	Three-tiered cut-off prognostic in p53-mutant and NSMP; PR 90–100% gave 5-year DSS of 100% in p53-mutant tumors [45]; no refinement within MMRd [22]	Not assessed against a comparator containing molecular class; in a pre-molecular comparison, the three-marker combination was only slightly better than histology [46]	III
L1CAM—adhesion, EMT phenotype; p53abn (80% expression) and NSMP	Strong association in unclassified cohorts [47,48,49,50,51,52]; independent of molecular subgroup in early-stage high-intermediate-risk disease [59]; not independent in the high-risk cohort [42]	Prognostic within NSMP [53,55]; pooled DSS HR 1.75 (1.06–2.89) in MMRd [54], not reproduced in 899 MMRd tumors [22]	One analysis, within 22 NSMP patients of a 47-patient external high-risk cohort: C-index 0.68 without and 0.75 with L1CAM after recalibration; neither a confidence interval nor a test of the difference reported; no comparison reported in the development cohort [55]	III, contested; IV in one small external NSMP subgroup only
*CTNNB1* exon 3 mutation—WNT activation; NSMP, low-grade early stage	Association reported [60,61]; not associated in the whole early-stage high-intermediate-risk group, but independent of molecular subgroup for distant recurrence in a selected subgroup [59]; not independent in the high-risk cohort [42]	DFS HR 2.83 (*p* = 0.026) restricted to NSMP and unclassified tumors [62], not a confirmed within-class analysis; no refinement within MMRd [22]	Not assessed for *CTNNB1* alone; included only within an integrated risk profile [59]	I, contested; II in a selected early-stage high-intermediate-risk subgroup only; pooled evidence does not demonstrate consistent prognostic significance [17]
*ARID1A*—chromatin remodeling; NSMP	Inconsistent association [16,17]; limited adjusted data	Worst outcome in *ARID1A*-mutant NSMP [63]; unfavorable effect confined to NSMP in meta-analysis [17]	Not assessed	III, contested
PI3K-pathway alterations—signaling; NSMP	Inconsistent association [17]; limited adjusted data	*PIK3CA* mutation associated with DSS within NSMP on univariable analysis [43]; no consistent effect of *PIK3CA*/*PTEN* mutations within NSMP in pooled analysis [17]	Not assessed	I, contested, for the pathway as a whole; III, contested, for *PIK3CA* mutation only
Chromosome 1q32.1 amplification—copy-number gain including *MDM4*; NSMP	Associated with recurrence-free and cancer-specific survival in NSMP-enriched copy-number clusters after adjustment for clinical covariates, but not for molecular class [64]	Recurrence-free survival HR 2.12 (1.26–3.59) in NSMP tumors of a discovery series; replicated in 487 NSMP tumors from PORTEC-1 and -2 (recurrence-free survival HR 2.34, 1.17–4.70; distant recurrence HR 3.98, 1.72–9.17); not prognostic in microsatellite-unstable or *TP53*-mutant tumors [64]	Not assessed; reported improvement in risk prediction based on survival differences between groups of a five-group classification [64]	III, replicated within NSMP
Genome-wide HRD score—genomic instability; assessed across classes	Associated with disease-free and overall survival after adjustment for age, stage and LVSI, but not for molecular class [67]; evaluated as an addition to TCGA classification	HRD subtypes separated outcomes within MSI-H and TP53 wild-type groups [67]	C-index for overall survival 0.751 (TCGA alone) vs. 0.903 (TCGA plus HRD); 0.669 vs. 0.848 for disease-free survival [67]	IV, internally demonstrated beyond TCGA classification alone
Intraepithelial CD8-positive T-cell density—immune microenvironment	Yes; multivariable Cox incorporating molecular class in 640 of 695 stage I tumors [69]	In exploratory analysis, effect greatest in p53-mutant and absent in MMRd [69]; lower density identified worse outcomes within MMRd in a separate cohort [22]	Comparison of multivariable models with internal bootstrap validation: molecular-immune model had the highest concordance; CD8 outweighed myoinvasion, grade and L1CAM [69]	IV, internally demonstrated
Semiquantitative LVSI—histopathology; all classes (comparator variable)	Established and independent	Focal and substantial prognostic in MMRd, only substantial in NSMP, neither significant in p53abn [13]	Not assessed as an addition to a molecular model	III
Biomarker	Clinical relevance		Main limitations	
ER	Sub-stratifies NSMP in the 2025 European risk groups, at a 10% threshold [10]; selects patients for the NSMP-directed RAINBO de-escalation trial [31]. Guideline use rests mainly on within-class evidence; clinical utility not yet demonstrated.		Thresholds differ (1%, 10%, three-tiered) [42,43,45] and there is no consensus cut-off [42]; largest NSMP series scored on tissue microarrays [43]; signal not reproduced within MMRd [22]; incremental performance measured in high-risk disease only and not externally validated [42].	
PR	Not used for risk grouping in the 2025 European guidelines [10]; supportive prognostic information only.		Correlated with ER, which carried the within-class signal in high-risk NSMP [42]; added value tested against a pre-molecular comparator only [46]; no refinement within MMRd [22].	
L1CAM	Not incorporated into the 2025 European risk grouping [10]. Component of the molecular-integrated risk profile used to allocate adjuvant treatment in PORTEC-4a, which tested the profile as a whole and not L1CAM individually (Section 7). Candidate modifier within NSMP in pooled analyses [16,17,54].		Concentrated in p53abn tumors, so unclassified associations partly reflect class [44,53]; not independent in high-risk disease after adjustment for clinicopathological factors and molecular class, or within high-risk NSMP, where the authors attributed this to its association with ER and PR negativity [42]; small within-class subgroups [55,56]; discordant within MMRd [22,54]; incremental performance tested only in 22 NSMP patients of one external cohort [55].	
*CTNNB1* exon 3 mutation	Not incorporated into the 2025 European risk grouping [10]; pooled evidence has not established consistent prognostic significance [17]. Component of the PORTEC-4a molecular-integrated risk profile, which was tested as a whole and not for *CTNNB1* individually (Section 7).		Signal reported mainly in low-grade, early-stage endometrioid disease [60,62]; molecularly adjusted association confined to a selected subgroup, with covariates selected in the same data [59]; no independent effect in high-risk disease [42]; no significant effect within NSMP in a further meta-analysis [16]; β-catenin immunohistochemistry an imperfect surrogate for mutation [61,62]; incremental performance not assessed.	
*ARID1A*	Investigational; possible adverse modifier within NSMP [17,63]. Not incorporated into the 2025 European risk grouping [10].		Few studies; the two recent meta-analyses disagree [16,17]; main supporting series comprised 125 tumors [63]; limited molecularly adjusted data; incremental performance not assessed.	
PI3K-pathway alterations	Investigational as a prognostic modifier.		No consistent prognostic effect of *PIK3CA*/*PTEN* alterations in pooled analysis [17]; limited molecularly adjusted data; incremental performance not assessed. The within-class association of *PIK3CA* mutation with DSS rests on one univariable analysis in NSMP [43] and does not extend to the pathway as a whole.	
Chromosome 1q32.1 amplification	Investigational; proposed as a refinement of NSMP [64]. Not incorporated into the 2025 European risk grouping [10]. Requires copy-number testing; no immunohistochemical surrogate identified [64].		Retrospective; cohorts differed in stage and assay (genome-wide arrays versus targeted probe amplification), and the amplified category combined focal and whole-arm events [64]; 1q copy-number status alone not prognostic in an NSMP series profiled by targeted sequencing [65]; incremental performance not assessed.	
Genome-wide HRD score	Hypothesis-generating: a large reported gain in discrimination when added to molecular class, from a single cohort [67].		Single center, *n* = 142, 18 recurrences, HRD-high cluster of 12 patients; subtypes and threshold derived in the cohort that tested them; comparator was molecular class alone; no external validation [67].	
Intraepithelial CD8-positive T-cell density	Investigational. Indicates that immune contexture can add to molecular class in stage I endometrioid disease; quantified by machine-learning image analysis rather than a routine assay [69].		Marker chosen from fifteen marker-compartment combinations in the cohort that tested it; internal bootstrap validation only; no calibration or decision-analytic evaluation [69]; distribution of the effect across classes disputed [22,69].	
Semiquantitative LVSI	Established risk factor embedded in FIGO 2023 staging and European risk grouping [9,10]; shows that prognostic meaning is class-dependent [13].		Single-center retrospective cohort; p53abn subgroup small (*n* = 62) and POLEmut not analyzable (one progression) [13]; not assessed as an addition to a molecular model.	

“Not assessed” means incremental predictive performance was not evaluated in the studies identified for this review, which is distinct from evidence that a marker adds nothing. The analyses behind the Level IV column, with their comparators, sample sizes and validation, are detailed in Supplementary Table S2, which also lists an analysis that added ER and L1CAM together with clinicopathological variables [66]. Clinical relevance describes current or proposed use and does not imply demonstrated clinical utility. No biomarker in the table has prospective evidence of clinical utility on its own; PORTEC-4a evaluated L1CAM and *CTNNB1* only as components of an integrated risk profile (Section 7). Levels were assigned with the rules set out in Section 3. A range (e.g., II–III) indicates that the higher level rests on limited evidence; “contested”, that analyses at that level reached discordant results; “replicated”, that a within-class result was confirmed in an independent cohort; “internally demonstrated”, that Level IV was shown without external validation. Other qualifiers give the setting or comparator to which a level applies. Guideline incorporation is listed under clinical relevance and does not by itself confer an evidence level. C-index, concordance index; DFS, disease-free survival; DSS, disease-specific survival; EMT, epithelial-to-mesenchymal transition; ER, estrogen receptor; FIGO, International Federation of Gynecology and Obstetrics; HR, hazard ratio; HRD, homologous recombination deficiency; L1CAM, L1 cell adhesion molecule; LVSI, lymphovascular space invasion; MMRd, mismatch repair-deficient; MSI-H, microsatellite instability-high; NSMP, no specific molecular profile; p53abn, p53-abnormal; PI3K, phosphoinositide 3-kinase; POLEmut, *POLE*-ultramutated; PR, progesterone receptor; TCGA, The Cancer Genome Atlas.

**Table 3:** Circulating biomarkers in molecularly classified endometrial cancer: evidence by level, clinical relevance and main limitations.

Biomarker	Molecular Adjustment	Within-Class Evidence (Level III)	Incremental Predictive Performance (Level IV)	Evidence Level Within This Review Framework
CA125, preoperative	Within-subtype analyses in one ProMisE-classified cohort [72]; retained in a multivariable model including ProMisE subgroup in another [81]	Associated with stage and nodal metastasis in each subtype; worse survival in p53abn, NSMP and MMRd [72]	Not assessed	III
HE4, preoperative	No molecular adjustment	Not reported	Not assessed	I
NLR	No molecular adjustment	Not reported	Not assessed	I
PLR	No molecular adjustment	Not reported	Not assessed	I
SII, MLR, CRP-based indices	No molecular adjustment; for MLR, no association with survival in pooled analysis [80], whose individual studies were discordant; SII and CRP-based indices not analyzed as individual markers in the studies cited	Not reported	Not assessed	I, contested for MLR only; no level assigned to SII or CRP-based indices (not analyzed as individual markers)
HALP score combined with ProMisE	Yes; molecular class is a model term [81]	Not analyzed by class	Nomogram assessed by C-index, calibration and decision curve, including in an external validation cohort, but not against the same model without the score [81]	II
Biomarker	Clinical relevance		Main limitations	
CA125, preoperative	Preoperative triage and risk enrichment for nodal or extra-uterine disease [72,73,74]. Not a stand-alone rule-in or rule-out test; not shown to improve a model that already contains molecular class.		Tracks tumor burden already captured by staging; limited accuracy for nodal metastasis [72]; within-subtype evidence from one molecularly classified cohort [72]; incremental performance not assessed.	
HE4, preoperative	Studied for prediction of nodal metastasis and monitoring of recurrence, alone or with CA125 [75,76,77]. No molecularly stratified data.		Renal function and age dependent; assay-specific thresholds; not evaluated within molecular classes; incremental performance not assessed.	
NLR	Inexpensive and available from routine blood counts; associated with survival in pooled analyses [79,80]. No role in molecularly informed risk stratification.		Cut-offs derived in the analysis dataset; low biological specificity; no adjustment for molecular class in either pooled analysis [79,80]; incremental performance not assessed.	
PLR	As for NLR, comparable pooled estimate for overall survival [79].		Overlaps with NLR; cut-offs vary between studies [79]; no molecular adjustment [79,80]; incremental performance not assessed.	
SII, MLR, CRP-based indices	Exploratory; no established clinical role.		Definitions and thresholds heterogeneous; no molecular adjustment; incremental performance not assessed.	
HALP score combined with ProMisE	Shows that an inflammatory-nutritional index can be modeled alongside molecular class, with discrimination, calibration and decision-curve analysis reported in an external validation cohort [81]; contribution of the score itself not isolated.		Retrospective; validated externally as a complete model only: not compared with an otherwise identical model omitting the score, so the incremental contribution of HALP remains untested [81].	

“Not assessed” means incremental predictive performance was not evaluated in the studies identified for this review. Clinical relevance describes current or proposed use and does not imply demonstrated clinical utility. Levels were assigned with the rules set out in Section 3 and are qualified as in Table 2. C-index, concordance index; CA125, cancer antigen 125; CRP, C-reactive protein; HALP, hemoglobin–albumin–lymphocyte–platelet; HE4, human epididymis protein 4; MLR, monocyte-to-lymphocyte ratio; MMRd, mismatch repair-deficient; NLR, neutrophil-to-lymphocyte ratio; NSMP, no specific molecular profile; p53abn, p53-abnormal; PLR, platelet-to-lymphocyte ratio; ProMisE, Proactive Molecular Risk Classifier for Endometrial Cancer; SII, systemic immune-inflammation index.

## Data Availability

No new data were created or analyzed in this study. Data sharing is not applicable to this article.

## Supplementary Materials

The following supporting information can be downloaded at https://www.mdpi.com/article/10.3390/jcm15208154/s1: Table S1: Assessment of this narrative review against the six items of the Scale for the Assessment of Narrative Review Articles (SANRA) [18]; Table S2: Analyses of incremental predictive performance among the studies cited in this review: comparator models, cohorts, events, results and validation.
