# Supplementary material for: Beyond Molecular Classification: The Incremental Value of Prognostic Biomarkers in Endometrial Cancer
Source: J Clin Med. 2026 Oct 20;15(20):8154. doi: 10.3390/jcm15208154 (PMC13706005; doi:10.3390/jcm15208154)
Supplement: Supplementary file 1 [file jcm-15-08154-s001.zip › jcm-4596368-supplementary.pdf]

Supplementary Materials

Beyond Molecular Classification: The Incremental Value of Prognostic Biomarkers in Endometrial Cancer

Table S1. Assessment of this narrative review against the six items of the Scale for the Assessment of Narrative Review Articles (SANRA) [18].

SANRA item	Rating (0–2)	Where addressed in the manuscript	Justification
1. Explanation of the review's importance for the readership	2	Abstract; Section 1, paragraphs 1–4	The Introduction explains why the question matters now: prognostic assessment decides adjuvant treatment in early-stage disease, molecular classification has entered the WHO classification, FIGO 2023 staging and European guidance, outcomes still vary within each class, and most biomarker studies did not test what a marker adds once molecular class is known.
2. Statement of the aims or questions of the review	2	Abstract; Section 1, final paragraph	A single question is stated: whether additional biomarkers provide prognostic information beyond contemporary molecular classification, across molecular classes and across tissue-based, circulating and emerging biomarker domains. The four evidentiary levels used to answer it, and the separate status of clinical utility, are named.
3. Description of the literature search	1	Section 1.1	The databases, the concepts combined in the searches, citation tracking, the evidence types given priority, the basis on which candidate biomarkers were discussed and the handling of discordant findings are described. Complete search strings and formal inclusion criteria are not given, in keeping with the narrative design, so the highest rating, which presupposes detailed search terms and inclusion criteria, is not claimed.
4. Referencing	2	Throughout the text; Tables 1–3	Key statements, including the entries supporting each evidence level in Tables 2 and 3, are referenced to the primary studies, meta-analyses, translational trial analyses, guidelines and methodological papers on which they rest.
5. Scientific reasoning	2	Sections 3–8	Claims are graded with an explicit framework that separates prognostic association, independence after adjustment for molecular class, within-class stratification and incremental predictive performance, with assignment rules stated and clinical utility kept apart. Discordant findings are presented alongside supportive ones, and the limitations of the evidence and of the review are stated. Coverage is purposeful rather than exhaustive (Section 7.1).
6. Presentation of relevant and appropriate endpoint data	2	Sections 4–6; Tables 2 and 3	Effect estimates are given with their precision where the source studies report it (hazard ratios with 95% confidence intervals; concordance indices before and after a marker is added; sensitivity and specificity), together with sample sizes, event numbers and the molecular class or setting to which each applies. A few estimates are reported as published, with a p-value rather than a confidence interval.
**Sum score**	**11 of 12**		Authors' assessment of the revised manuscript.

Ratings follow the SANRA scale, on which each item is rated 0 (low standard), 1 (intermediate) or 2 (high standard), giving a sum score of 0–12 [18]. The ratings are the authors' assessment of the revised manuscript; SANRA has no established cut-offs for grading overall quality [18]. SANRA is a scale for appraising the quality of narrative review articles; its use here does not imply a systematic literature search, formal study selection or risk-of-bias assessment, none of which was part of this narrative review.

Reference

18. Baethge, C.; Goldbeck-Wood, S.; Mertens, S. SANRA—A scale for the quality assessment of narrative review articles. *Res. Integr. Peer Rev.* **2019**, *4*, 5. https://doi.org/10.1186/s41073-019-0064-8

Table S2. Analyses of incremental predictive performance among the studies cited in this review: comparator models, cohorts, events, results and validation.

Study; marker or variables added	Population and design	Patients, events and follow-up	Molecular classification	Comparator model	Outcome; measure and result (comparator vs. comparator plus marker)	Calibration; reclassification	Validation	Main limitations
Comparison of the same model with and without the added marker or set of variables								
Vermij et al., 2023 [42]; ER (10% threshold) dividing NSMP into ER-positive and ER-negative	High-risk EC; PORTEC-3 trial (n = 411) and a Dutch prospective cohort treated with adjuvant radiotherapy in 1987–2015 (n = 237)	648 (models: 643; 207 events); median follow-up 7.0 years	MMR and p53 IHC on whole slides (MSI assay or *TP53* sequencing if IHC failed); *POLE* hotspot sequencing; four classes	Multivariable Cox model from backward selection, stratified by cohort: age >60 years, stage, histotype and grade, LVSI, molecular class (four classes), adjuvant treatment (chemoradiotherapy vs. radiotherapy)	Recurrence-free survival. AIC 2173.77 vs. 2162.38; C-index 0.712 vs. 0.726; likelihood ratio test $P < 0.001$	NR; NR	Internal (1000 bootstrap resamples; leave-one-cohort-out re-estimation), reported for the ER effect within NSMP; no external validation	Authors: no external validation; 10% threshold to be validated; lower-risk NSMP not studied. Review: ER division identified in the same cohort; small gain; high-risk disease only
Ravaggi et al., 2022 [55]; L1CAM (>10% of tumor cells) dividing NSMP into L1CAM-positive and L1CAM-negative	High-risk EC (high-intermediate, high or advanced-metastatic risk class); single centre, retrospective, 2004–2019; models applied to an external ENITEC cohort	Development: 94; 50 recurrences or progressions; median follow-up 81.3 months. External: 47 (NSMP, 22; 18% of NSMP patients relapsed or died of disease)	*POLE* exonuclease-domain sequencing; IHC for PMS2, MSH6 and p53; multiple classifiers assigned by published rules; four classes	Survival model without L1CAM, described by the authors as based only on molecular classification; extended model: the same model with L1CAM added. Covariates of the validated models not listed; the development multivariable model included age, stage and grade	Progression-free survival. Within NSMP of the external cohort, after recalibration: C-index 0.68 vs. 0.75; CI and test of the difference NR. No C-index reported for the development cohort	Logistic recalibration. Predicted 36-month PFS in L1CAM-positive and L1CAM-negative NSMP: 0.63 and 0.66 with L1CAM, 0.66 and 0.64 without; observed 0.75 and 0.90. Reclassification NR	Comparison made only in the external cohort (47 patients; 22 NSMP), after recalibration of the models to that cohort	Authors: small cohort; confirmation in wider cohorts needed. Review: limited evidence from one externally tested comparison in 22 patients with few events, not validation of an established incremental effect
Zagidullin et al., 2022 [66]; ER and L1CAM status, peritoneal washing status and tumor diameter >5 cm, added together	Unselected EC; single centre, retrospective; surgery 2007–2012	700 of 842 analyzed (MMRd 305, NSMP 308, p53abn 87); 182 recurrences and 147 deaths (deaths from other causes excluded); p53abn: 41 deaths; median follow-up 82 months (whole cohort)	ProMisE, assigned experimentally in 604 of 842 (71.7%) and imputed for the remainder; POLEmut excluded from the analysis	Feature set I: age, FIGO stage, histological subgroup, ProMisE class (constant within subgroup models), deep myometrial invasion, LVSI, tumor diameter >3 cm; variables selected in the same data	Disease-specific survival. p53abn (n = 87): C-index 0.764 vs. 0.774 (Cox model), 0.725 vs. 0.794 (optimal survival tree). MMRd (n = 305), tree: 0.789 vs. 0.884. Whole cohort, tree: 0.849 vs. 0.859. NSMP (n = 308): highest C-index with the Cox model without the added set (0.838). Among Cox models, largest gain in p53abn	Integrated Brier score (discrimination and calibration) improved by 3% (Cox) and 19% (tree) in p53abn; reclassification NR	Bootstrap confidence intervals; no external validation	Authors: no external validation cohort; p53abn Cox model not advised for interpretation-dependent use such as nomograms. Review: variables selected and performance estimated in the same data (bootstrap intervals only); flexible tree models prone to overfitting; variables added as a set, so the contribution of ER or L1CAM cannot be separated

Study; marker or variables added	Population and design	Patients, events and follow-up	Molecular classification	Comparator model	Outcome; measure and result (comparator vs. comparator plus marker)	Calibration; reclassification	Validation	Main limitations
Wang et al., 2026 [67]; HRD subtypes (high, middle, low) derived by clustering of genomic scar scores	EC, FIGO stage I–IV; single centre; surgery 2011–2020	142; 18 relapses; deaths NR; median follow-up 38.8 months	NGS-based TCGA surrogate (*POLE* exonuclease-domain mutation, MSI by sequencing, *TP53* mutation); four groups	TCGA classification alone; no clinicopathological variables	Overall survival: C-index 0.751 vs. 0.903 (HRD subtypes alone 0.857). Disease-free survival: 0.669 vs. 0.848 (HRD subtypes alone 0.787)	NR; NR	None reported for the model comparison	Authors: small sample; thresholds from maximally selected rank statistics. Review: subtypes and threshold derived in the cohort that tested them; HRD-high cluster of 12 patients; comparator without clinicopathological variables
Horeweg et al., 2020 [69]; intraepithelial CD8-positive cell density (continuous, per doubling)	Stage I endometrioid EC from the PORTEC-1 and PORTEC-2 trials; tissue microarrays with machine-learning quantification	695 (models: 640; 80 recurrences, 53 cancer-specific deaths); median follow-up 11.2 years	*POLE* hotspot sequencing; MMR and p53 IHC; four classes	Prespecified molecular model: myometrial invasion, grade, LVSI, molecular class and L1CAM	Recurrence: AIC 842.2 vs. 835.4; C-index 0.697 vs. 0.726; likelihood ratio test P = 0.003. Cancer-specific survival: AIC 544.9 vs. 541.4; C-index 0.792 vs. 0.802; P = 0.019	NR; NR	Internal (1000 bootstrap resamples: discrimination and optimism); no external validation	Authors: tissue microarrays; exploratory subgroup findings require validation; the main finding is to be validated, particularly in higher-risk cohorts. Review: marker selected from fifteen marker-compartment combinations in the same cohort
Comparison of a separate integrated model with a comparator containing molecular class (not a comparison of the same model with and without a marker)								
Volinsky-Fremond et al., 2024 [68]; HECTOR deep learning model (H&E whole-slide image, molecular class predicted from the image, stage)	FIGO 2009 stage I–III EC from eight cohorts, including the PORTEC-1, -2 and -3 trials; development with held-out internal and external test sets	Training 1408 (246 distant recurrences; median follow-up 7.8 years); internal test 353 (62; 8.4 years); external tests 160 (14; 5.3 years) and 151 (24; 2.9 years)	Comparator: molecular class from sequencing and IHC, missing class imputed by mean substitution. HECTOR: class predicted from H&E	Cox model with histological subtype, grade, LVSI, stage and molecular class	Distant recurrence. Five-fold cross-validation in the training set: C-index 0.762 (95% CI 0.732–0.791) vs. 0.795 (0.768–0.822) for HECTOR. Test sets, HECTOR: 0.789 internal; 0.828 and 0.815 external (0.815 with up to three slides per patient)	Cumulative AUC and integrated Brier score reported by the authors in a supplementary table; calibration plots and reclassification against the comparator NR in the article text	Comparison with the comparator reported from cross-validation in the development data; HECTOR discrimination reported in held-out internal and external test sets	Authors: validation needed in more diverse cohorts and prospective studies. Review: a different model rather than a marker added to the comparator; comparator performance in the external test sets not reported in the article text

The table lists the analyses, among the studies cited in this review, that compared a model containing molecular class, or fitted within one molecular class, with the same model after a marker or a set of variables had been added (upper block), and one analysis that compared a separate integrated model with such a model (lower block). It is not the result of a systematic search. Data were extracted from the full texts of the tabulated studies. Analyses without such a comparison are not included, among them a comparison with a comparator that did not contain molecular class [46] and an externally validated nomogram that was not compared with the same model without its marker [81]. Two analyses that reported improved risk prediction are not tabulated, because their articles give no numerical comparison of model performance. In the PORTEC-1 and -2 trials, areas under the curve for the molecular-integrated risk profile and for molecular classification were presented only graphically [59]. A five-group classification that separated 1q32.1-amplified NSMP tumors was supported by survival differences between the groups rather than by a measure of model performance against the four-class classification [64]. Neither omission implies that the integrated profile or the marker lacks incremental value. Results are given as comparator vs. comparator plus marker unless stated otherwise. Limitations are those stated by the investigators (Authors) and those noted in this review (Review). NR, not reported in the article text examined.

AIC, Akaike information criterion; AUC, area under the curve; CI, confidence interval; C-index, concordance index; EC, endometrial cancer; ENITEC, European Network for Individualized Treatment of Endometrial Cancer; ER, estrogen receptor; FIGO, International Federation of Gynecology and Obstetrics; H&E, hematoxylin and eosin; HRD, homologous recombination deficiency; IHC, immunohistochemistry; L1CAM, L1 cell adhesion molecule; LVSI, lymphovascular space invasion; MMR, mismatch repair; MMRd, mismatch repair-deficient; MSI, microsatellite instability; NGS, next-generation sequencing; NSMP, no specific molecular profile; p53abn, p53-abnormal; PFS, progression-free survival; POLEmut, *POLE*-ultramutated; ProMisE, Proactive Molecular Risk Classifier for Endometrial Cancer; TCGA, The Cancer Genome Atlas.

References

42. Vermij, L.; Jobsen, J.J.; León-Castillo, A.; Brinkhuis, M.; Roothaan, S.; Powell, M.E.; de Boer, S.M.; Khaw, P.; Mileshkin, L.R.; Fyles, A.; et al. Prognostic refinement of NSMP high-risk endometrial cancers using oestrogen receptor immunohistochemistry. *Br. J. Cancer* **2023**, *128*, 1360–1368. https://doi.org/10.1038/s41416-023-02141-0
46. van der Putten, L.J.M.; Visser, N.C.M.; van de Vijver, K.; Santacana, M.; Bronsert, P.; Bulten, J.; Hirschfeld, M.; Colas, E.; Gil-Moreno, A.; Garcia, A.; et al. Added value of estrogen receptor, progesterone receptor, and L1 cell adhesion molecule expression to histology-based endometrial carcinoma recurrence prediction models: An ENITEC collaboration study. *Int. J. Gynecol. Cancer* **2018**, *28*, 514–523. https://doi.org/10.1097/IGC.0000000000001187
55. Ravaggi, A.; Capoferri, D.; Ardighieri, L.; Ghini, I.; Ferrari, F.; Romani, C.; Bugatti, M.; Zanotti, L.; Vrede, S.; Tognon, G.; Pijnenborg, J.M.A.; Sartori, E.; Calza, S.; Bignotti, E.; Odicino, F. Integrated biomarker analysis reveals L1CAM as a potential stratification marker for no specific molecular profile high-risk endometrial carcinoma. *Cancers* **2022**, *14*, 5429. https://doi.org/10.3390/cancers14215429
59. Stelloo, E.; Nout, R.A.; Osse, E.M.; Jürgenliemk-Schulz, I.J.; Jobsen, J.J.; Lutgens, L.C.; van der Steen-Banasik, E.M.; Nijman, H.W.; Putter, H.; Bosse, T.; et al. Improved risk assessment by integrating molecular and clinicopathological factors in early-stage endometrial cancer—Combined analysis of the PORTEC cohorts. *Clin. Cancer Res.* **2016**, *22*, 4215–4224. https://doi.org/10.1158/1078-0432.CCR-15-2878
64. Depreeuw, J.; Stelloo, E.; Osse, E.M.; Creutzberg, C.L.; Nout, R.A.; Moisse, M.; Garcia-Dios, D.A.; Dewaele, M.; Willekens, K.; Marine, J.-C.; et al. Amplification of 1q32.1 refines the molecular classification of endometrial carcinoma. *Clin. Cancer Res.* **2017**, *23*, 7232–7241. https://doi.org/10.1158/1078-0432.CCR-17-0566
66. Zagidullin, B.; Pasanen, A.; Loukovaara, M.; Bützow, R.; Tang, J. Interpretable prognostic modeling of endometrial cancer. *Sci. Rep.* **2022**, *12*, 21543. https://doi.org/10.1038/s41598-022-26134-w
67. Wang, W.; Gao, M.; Gao, Y.; Zheng, H.; Song, N.; Zhang, N.; Shu, T.; Gao, W.; Zhang, N.; Wang, H. Integrating homologous recombination deficiency subtyping with TCGA molecular classification for enhanced prognostic stratification and personalised therapy in endometrial cancer. *Br. J. Cancer* **2026**, *134*, 119–130. https://doi.org/10.1038/s41416-025-03179-y
68. Volinsky-Fremond, S.; Horeweg, N.; Andani, S.; Barkey Wolf, J.; Lafarge, M.W.; de Kroon, C.D.; Ørtoft, G.; Høgdall, E.; Dijkstra, J.; Jobsen, J.J.; Lutgens, L.C.H.W.; Powell, M.E.; Mileshkin, L.R.; Mackay, H.; Leary, A.; Katsaros, D.; Nijman, H.W.; de Boer, S.M.; Nout, R.A.; de Bruyn, M.; Church, D.; Smit, V.T.H.B.M.; Creutzberg, C.L.; Koelzer, V.H.; Bosse, T. Prediction of recurrence risk in endometrial cancer with multimodal deep learning. *Nat. Med.* **2024**, *30*, 1962–1973. https://doi.org/10.1038/s41591-024-02993-w
69. Horeweg, N.; de Bruyn, M.; Nout, R.A.; Stelloo, E.; Kedzierska, K.; León-Castillo, A.; Plat, A.; Mertz, K.D.; Osse, M.; Jürgenliemk-Schulz, I.M.; et al. Prognostic integrated image-based immune and molecular profiling in early-stage endometrial cancer. *Cancer Immunol. Res.* **2020**, *8*, 1508–1519. https://doi.org/10.1158/2326-6066.CIR-20-0149
81. Zheng, Y.; Yang, F.; Liu, G.; Wu, X.; Xie, L.; Hu, R.; Luo, X.; Yuan, R. Development and validation of a prediction model for recurrence based on ProMisE molecular classifier and immune-inflammatory-nutritional score in endometrial carcinoma: A retrospective multiple-center study. *Int. J. Med. Sci.* **2025**, *22*, 3789–3801. https://doi.org/10.7150/ijms.107134
